# Supplementary material for: Opportunities and challenges for decentralised clinical trials in sub-Saharan Africa: a qualitative study
Source: BMJ Open. 2023 Sep 22;13(9):e075903. doi: 10.1136/bmjopen-2023-075903 (PMC10533674; doi:10.1136/bmjopen-2023-075903)
Supplement: Author's manuscript [file bmjopen-2023-075903.draft_revisions.pdf]

BMJ Open

BMJ Open is committed to open peer review. As part of this commitment we make the peer review history of every article we publish publicly available.

When an article is published we post the peer reviewers' comments and the authors' responses online. We also post the versions of the paper that were used during peer review. These are the versions that the peer review comments apply to.

The versions of the paper that follow are the versions that were submitted during the peer review process. They are not the versions of record or the final published versions. They should not be cited or distributed as the published version of this manuscript.

BMJ Open is an open access journal and the full, final, typeset and author-corrected version of record of the manuscript is available on our site with no access controls, subscription charges or pay-per-view fees (http://bmjopen.bmj.com).

If you have any questions on BMJ Open's open peer review process please email info.bmjopen@bmj.com

BMJ Open

Investigators, Contract Research Organisations, and Sponsors' Perspectives of Digitalised and Decentralised Clinical Trials in Sub-Saharan Africa

Journal:	*BMJ Open*
Manuscript ID	bmjopen-2023-075903
Article Type:	Original research
Date Submitted by the Author:	22-May-2023
Complete List of Authors:	Nebie, Eric; Centre de Recherche en Sante de Nouna, ; Swiss TPH, Medicines Implementation Research Unit<br>Sawadogo, Hélène N.; Centre de Recherche en Santé de Nouna<br>van Eeuwijk, Peter; Swiss Tropical and Public Health Institute; University of Basel<br>Signorell, Aita ; Swiss Tropical and Public Health Institute; University of Basel<br>Reus, Elisabeth; Swiss Tropical and Public Health Institute; University of Basel<br>Utzinger, Juerg; Swiss Tropical and Public Health Institute; University of Basel<br>Burri, Christian; Swiss Tropical and Public Health Institute; University of Basel
Keywords:	Clinical Trial, QUALITATIVE RESEARCH, PUBLIC HEALTH

SCHOLARONE™
Manuscripts

I, the Submitting Author has the right to grant and does grant on behalf of all authors of the Work (as defined in the below author licence), an exclusive licence and/or a non-exclusive licence for contributions from authors who are: i) UK Crown employees; ii) where BMJ has agreed a CC-BY licence shall apply, and/or iii) in accordance with the terms applicable for US Federal Government officers or employees acting as part of their official duties; on a worldwide, perpetual, irrevocable, royalty-free basis to BMJ Publishing Group Ltd ("BMJ") its licensees and where the relevant Journal is co-owned by BMJ to the co-owners of the Journal, to publish the Work in this journal and any other BMJ products and to exploit all rights, as set out in our licence.

The Submitting Author accepts and understands that any supply made under these terms is made by BMJ to the Submitting Author unless you are acting as an employee on behalf of your employer or a postgraduate student of an affiliated institution which is paying any applicable article publishing charge ("APC") for Open Access articles. Where the Submitting Author wishes to make the Work available on an Open Access basis (and intends to pay the relevant APC), the terms of reuse of such Open Access shall be governed by a Creative Commons licence – details of these licences and which Creative Commons licence will apply to this Work are set out in our licence referred to above.

Other than as permitted in any relevant BMJ Author's Self Archiving Policies, I confirm this Work has not been accepted for publication elsewhere, is not being considered for publication elsewhere and does not duplicate material already published. I confirm all authors consent to publication of this Work and authorise the granting of this licence.

For peer review only

Investigators, Contract Research Organisations, and Sponsors' Perspectives of Digitalised and Decentralised Clinical Trials in Sub-Saharan Africa

Eric Ipyn Nebié[1,2,3], Hélène N. Sawadogo[3], Peter van Eeuwijk[1,2,4], Aita Signorell[1,2], Elisabeth Reus[1,2], Jürg Utzinger[1,2] and Christian Burri[1,2]

[1]Swiss Tropical and Public Health Institute, Kreuzstrasse 2, CH–4123 Allschwil, Switzerland

[2]University of Basel, P.O. Box, CH–4003 Basel, Switzerland

[3]Centre de Recherche en Santé de Nouna, Nouna, Burkina Faso

[4]Institute of Social Anthropology, University of Basel, Basel, Switzerland

For peer review only

Key message

- **What is already known on this topic:** The benefits of the digitalization and decentralization of clinical trials (DDCTs) are still controversial despite the increased uptake of DDCTs during the Covid 19 pandemic.
- **What this study adds:** DDCTs are feasible and perceived as an opportunity for clinical trials efficiency improvement in sub-Saharan Africa despite the challenges for their implementation. DDCTs ensured the continuity of clinical trials during the Covid 19 pandemic in sub-Saharan Africa clinical research sites. However, this patient centric transformation should be context specific and should not waive the benefits of current procedures in sub-Saharan Africa nor the responsibilities of investigators and sponsors.
- **How this study might affect research, practice or policy:** Careful planning and site assessment are necessary for an efficient DDCTs transition. The digital transformation in clinical trials should be partial, and requires the buy-in of ethic and regulatory authorities as well as the involvement of investigators and potential trial participants in the process.

For peer review only

Abstract

Introduction

Digital health has gained traction in research and clinical decision support systems. The COVID-19 pandemic accelerated the adoption of digitalised and decentralised clinical trials (DDCTs) as a mitigation and efficiency improvement strategy. We assessed investigators, contract research organisations, and sponsors' perspectives on the digital transformation in clinical research in sub-Saharan Africa.

Methods

A qualitative study including 40 in-depth semi-structured interviews was conducted with investigators of three leading research institutions in sub-Saharan Africa and Switzerland, contract research organisation, and sponsors managing clinical trials in sub-Saharan Africa. The data were inductively and iteratively analysed using a thematic coding approach. The findings grounded on methodological triangulation with direct observations and documents review.

Results

DDCTs were perceived as feasible and providing an opportunity for site empowerment, trial efficiency improvement, and reducing trial burden on participants. However, to gain and maintain an optimal quality of trials, a transition period is necessary to tackle contextual challenges before DDCTs implementation. The main challenges are categorised into four themes: (i) context specific challenges; (ii) technology suitability and mastering; (iii) ethical and regulatory hurdles; and (iv) adaptability to the changing paradigm.

Conclusion

The transformation from a site to patient centric model with an increased responsibility of participants should be adjusted to the local context. This requires financial investment, training, communication, and community engagement. Additionally, commitment of sponsors, investigators, ethics, and regulatory authorities and the buy-in of the communities are essential for a successful and lasting digital shift.

Keywords: Clinical research; clinical trials; digital health; digitalised and decentralised clinical trials; sub-Saharan Africa

For peer review only

1. Introduction

Digital health technologies are prominent innovations in global health as they hold promise to improve routine health care and foster the implementation of new health interventions (1). The concept of digitalised and decentralised clinical trials (DDCTs) is participant-centred (2) and involves the use of digital technologies in the continuum of care and research from community engagement, participants enrolment, trial conduct, and management to the reporting. DDCTs are being developed as a promising solution for future clinical research (3). DDCTs aim to address the productivity crisis in drug development with continuously increasing costs, duration, burden, and complexity of clinical trials (4, 5). The first documented exclusive DDCT (i.e., without any participant visit to the clinic), was the "REMOTE trial", which provided the basis for future digital and decentralized clinical trials (6). Prior to the COVID-19 pandemic, electronic data capture (EDC), electronic health records, and telemedicine were the main digitalised areas in clinical trials. The use of wearable devices for electronic patient reported outcome (ePRO) and electronic clinical outcome assessment (eCOA), as well as artificial intelligence (AI) is still at an early adoption stage in clinical trials due to the scarcity of regulatory guidelines. The COVID-19 pandemic forcedly shed light on the opportunities for fast-track medical product development, while ensuring continuity of trials under the pandemic's stringent restrictions (7). DDCTs have proven to be feasible and to have potential to improve participant recruitment, retention, and representativity in clinical trials (8, 9), data collection (10), and efficiency (11). However, in a recent systematic review from studies conducted in Asia, Australia, Europe, and North America, there was little consensual added value of digitalisation in clinical research (3).

Before the COVID-19 pandemic, DDCTs were not fully covered by ethics and regulatory guidelines. Guidance on electronic records and electronic signatures were first included into the US Code of Federal Regulations Title 21, Part 11 (21 CFR Part 11). The first version of the International Conference on Harmonization (ICH) Guideline E6 "Good Clinical Practices" of 1996 (ICH E6 GCP) (12) mentioned electronic data collection in a few instances; yet, at a rather superficial way. By February 2022, only two countries (Denmark and Switzerland) had digital clinical trials included in their guidelines (13). In-depth deliberation of the topic was initiated with a European Medicines Agency (EMA) position paper covering electronic source data and data transcribed to electronic data collection tools and further detailed in the EMA recommendation paper in December 2022 (14). The ICH E6 GCP revision R2, put forth in 2016 (12), mentioned computerized systems, but still in a fragmented way with particular reference to systems validation and risk management and with little specificity. Hence, the adoption of these changes was challenging. Of note, ICH E6 GCP is currently updated to R3. First draft documents revealed that digital tools will receive more attention and the integration of novel

For peer review only - http://bmjopen.bmj.com/site/about/guidelines.xhtml

technology is foreseen. The validation of systems, electronic consent, data protection, information technology (IT) security, and user management are key elements mentioned (12).

In sub-Saharan Africa, the transition to DDCTs is still at an earliest stage. With the outset and rapid spread of SARS-CoV-2, DDCTs were expeditiously initiated in research institutions in sub-Saharan Africa to allow continuity of ongoing clinical trials despite the unfolding pandemic. The current study aimed to address the opportunities and challenges for an efficient transition to DDCTs, placing particular emphasis on sub-Saharan Africa.

2. Methods

2.1. Study Setting

Data were collected in two leading clinical research institutions in sub-Saharan Africa; the Malaria Research and Training Centre (MRTC) in Mali and the Clinical Research Unit of Nanoro (URCN) in Burkina Faso. Additionally, data were obtained from four departments of the University Hospital Basel (USB) in Switzerland, which are conducting clinical trials. The USB is also involved in clinical trials conducted in various settings in sub-Saharan Africa.

The two African research institutions have a long-standing clinical research experience with various sponsors from academia, product development partnerships (PDPs), and the pharmaceutical industry. These centres have strong capacities in terms of infrastructures, equipment, and human resources and comply with international standards (15). The two research institutions' catchment areas include both rural and semi-urban settings.

2.2. Study Design

The main aim of the study was to assess the efficiency of drug development by comparing the approaches of investigator-initiated trials, PDPs, and the pharmaceutical industry in terms of study operations, costs, and duration of clinical trials (16). We pursued a qualitative study, embedded in a sequential exploratory mixed method design in which a qualitative study component informed the development of the quantitative component. A 6-week work shadowing in each of the three purposively selected research institutions (MRTC, CRUN, and USB) was performed to better understand study operations processes.

2.3. Direct Observation

We compared the organisation and processes of clinical trials implementation according to sponsor type. During the work shadowing, non-participatory, yet direct observation of the site's ongoing studies operations and management was performed using predefined observation guidelines. Contextual information was recorded in a "field journal" (17) and was summarised.

For peer review only - http://bmjopen.bmj.com/site/about/guidelines.xhtml

For peer review only

2.4. Documentation Review

Two completed or ongoing Phase II or Phase III trials from two different sponsors (academia, PDP, or pharma) were selected in each site for review of the documentation, i.e., the investigator site file and/or the trial master files and ancillary documents (field activities planning, project management tools), and other general sites management documentation in electronic and printed form.

2.5. Interviews

The in-depth semi-structured interviews were conducted with clinical researchers (principal investigators and study coordinators), sponsors (project managers, project leaders, clinical development staff, quality and regulatory affairs responsibles, and medical officers) and contract research organisations (CROs) managers who have been involved in at least one clinical trial in the past 3 years in sub-Saharan Africa.

A scientific advisory committee, comprising representatives from each sponsor type and a sub-Saharan Africa research institution leader, reviewed all the tools (interview guide, document review guide and work shadowing tools). After their input, all instruments were pilot tested.

2.6. Data Collection and Analysis

The work shadowing took place from December 2020 to October 2021. Interviews were either performed face-to-face or virtually using a Zoom license maintained by Swiss TPH. The interviews were recorded, transcribed, coded, and analysed following an inductive and iterative process. The coding was performed by two researchers who assessed the transcripts and performed a thematic analysis using MAXQDA version 20 (VERBI Software 2021; Berlin, Germany). The subcodes and codes were generated and grouped into categories and themes. The trial operations, particularly informed consent, participant recruitment, site organisation, decision-making process, data collection, and management, were scrutinised to deepen the understanding of the research context. Paper versions of the available documentation on-site were directly assessed, and information was triangulated with the findings of the interviews.

2.7 Patient and public involvement statement

We did not include patients in the design, conduct, reporting and dissemination plan of the study. The scientific advisory committee comprising representatives from each sponsor type and a sub-Saharan African investigator was involved in the design, the conduct and the reporting of the study. The committee provided insights to all the tools before data collection.

For peer review only - http://bmjopen.bmj.com/site/about/guidelines.xhtml

3. Results

Overall, 40 in-depth semi-structured interviews were conducted. Previous experience of DDCTs before and after the onset of the COVID-19 pandemic, perceived context-specific DDCTs opportunities and challenges, and potentials solutions were discussed during the interviews. The interviewees' characteristics are summarised in Table 1.

Table 1: Interviewees' Characteristics

Participants' profiles		Number
**Gender**		
	**Male**	29
	**Female**	11
	**Total**	40
**Country of residence**		
	**Burkina Faso**	8
	**Germany**	2
	**Kenya**	1
	**Mali**	9
	**Senegal**	1
	**South Africa**	2
	**Switzerland**	15*
	**USA**	1
	**Total**	40
**Main role**		
	**CRO managers**	4
	**Principal investigators**	12
	**Sponsor PDPs**	6
	**Sponsors Pharma**	6
	**Study coordinators**	12
	**Total**	40
**Experience in clinical research**		
	**<5 years**	6
	**5 -10 years**	12
	**≥10 years**	22
	**Total**	40
**Mode of interviews**		
	**Face-to-face**	21
	**Online**	19
	**Total**	40

**Respectively seven and eight from the sponsors and the University Hospital Basel (USB).*

3.1. Previous Experience with Digital Clinical Trials

The sites were supported by community health workers and key informants for community engagement, participants' recruitment, and follow-up through home visits. Electronic data capture, telephone calls, and home visits were the main digital and decentralised solutions used in the visited sites. All the assessed studies were using electronic data capture tools. In turn, none of the interviewees in sub-Saharan African sites had so far used wearable sensor devices for remote monitoring of trials participants as evidenced by the following statement: *"Basically, we are working with fieldworkers who perform home visits when we do not want to move the participant to the site" (male, investigator, sub-Saharan Africa).*

3.2. Perceptions Regarding the Use of DDCTs

For peer review only - http://bmjopen.bmj.com/site/about/guidelines.xhtml

The perceptions of most of the interviewees on the potential innovation of DDCTs were mixed, and hence, should be interpreted in context. Although most of the investigators and sponsors interviewed perceived DDCTs as an asset for clinical research in sub-Saharan Africa, they stressed the need to address the challenges for their adoption and implementation.

A senior investigator, for instance, shared the perception about the fundamental applicability and feasibility of DDCTs in sub-Saharan Africa as follows: *“It's a matter of explanation – with social media, things evolve faster. Here, it is quite feasible; we have to plan a backup at the beginning of the process”* (male, principal investigator).

The same perception is shared by another senior investigator, stressing the ability of sites to adjust to new technologies provided that there is sufficient information about the new tools as this was the case for electronic data capture: *“We have been able to collect data electronically, and we have not had any problems. We can adapt in the same way as we have adapted to e-CRFs and others”* (male, principal investigator).

Investigators suggested that reflection on digitalisation of clinical trials should start now and mature before the full deployment of DDCTs. Furthermore, some investigators expressed that changes should be limited and that they would prefer the conventional clinical trial operations. They further suggested careful and timely planning for a consistent digital transformation. *“After COVID-19, we should go back to the old system, we should not digitalise everything”* (male, study coordinator).

3.3. Opportunities for DDCTs

3.3.1. Site Empowerment and Sustained Site-Sponsor Collaboration

DDCTs offered the possibility to pursue ongoing clinical trials especially during the COVID-19 pandemic. Despite travel restrictions during the pandemic, the visited sites did not pause their ongoing trials for long periods (less than 6 months). Owing to their experience in clinical research with the sponsors, the sites received a shift of more delegated tasks allowing them to take more responsibilities within the trials. This experience was perceived as successful as shared by a principal investigator: *“COVID-19 has ensured that this development [DDCTs] is moving forward and we should explore and also move forward in this area. We have already started, and it is very successful. For example, in this study of [compound] conducted with [pharmaceutical company], that we completed- it was all virtual. [...] we do not have any difference. The sponsor knows our institution already – so, for experienced centres, it remains a possibility”* (male, principal investigator).

The remote monitoring visits were much appreciated by the investigators during the COVID-19 pandemic and were perceived as efficient as the conventional approach. During the work shadowing, we witnessed a closeout visit and discussed the process with the investigators.

For peer review only - http://bmjopen.bmj.com/site/about/guidelines.xhtml

3.3.2. Improved Time and Cost-Efficiency

DDCTs were perceived as an efficient approach for CROs, investigators, and sponsors for cost optimisation and timesaving. The increased adoption of virtual meetings, training activities as well as mix- or centralised monitoring during the COVID-19 pandemic reinforced a need of change in current trial operations. A senior CRO manager particularly mentioned the time, energy, and cost savings through travel reduction *“Some of the training could be done virtually and save time on the spot, probably save some travel, so there is definitely a cost-efficiency that can be real”* (male, CRO manager).

However, these perceptions are controversial for some sponsors and investigators arguing the potential drawbacks of virtual meetings and trainings, stressing that there is no real overall “cost-saving" since more investments will be needed to finalise the trial due to its poor quality and downturn of experience sharing opportunities, as mentioned by a senior sponsor representative. *“You can conduct trainings and meetings virtually, but you also loose the experience sharing. It does not seem that it would be less expensive because you do not have to pay for the flights to these people, but in the end, it will not decrease the costs of the study because it will delay the reporting”* (male, sponsor staff).

3.3.3. Trial Burden Reduction

Investigators and sponsors supported that DDCTs could decrease the burden of trials on participants in terms of travel and time, particularly for participants living in difficult to reach areas. This could improve the geographic diversity in participants’ recruitment. *"It can be a way to reduce the burden on patients in trials. They don’t have to come to the site, five times.”* (female, sponsor staff).

3.3.4. Trial Quality and Documentation Improvement

All interviewed sponsors perceived DDCTs as an opportunity to open the discussion on innovation in clinical research, especially after the lessons learned from trials conducted during the COVID-19 pandemic. The interviewees stressed the need of innovative tools to ensure trial quality and considered DDCTs as a promising option. *“There will be lessons to be learned, especially about the way we conduct trials, and we may have to think about new ways of ensuring quality"* (female, sponsor representative).

Moreover, digitalisation allows remote and real-time access to the data and timely corrective measures if need be. This perception is also shared by CRO managers to speed-up trial monitoring. Digitalised documentation, e-CRF, and trial files (investigator site file and trial master files) facilitated the continuity of clinical trials, even during the COVID-19 pandemic and reduced the logistic burden of the files being shipped from or to the sponsor. Three

For peer review only - http://bmjopen.bmj.com/site/about/guidelines.xhtml

assessed studies successfully changed their master files from paper-based to electronic in the sites. *"Being able to do an eCRF and electronic trial master file improves efficiency. It might be a bit more expensive than a paper set-up, but it helps because you get your data in-house faster. I think there's a positive link between technology and clinical trials, and it can only get better"* (male, sponsor representative).

However, the consistent digitalisation of all the processes of clinical trials could be burdensome and become a quality issue if various electronic platforms and systems were used during the trials, as stressed by a sponsor's quality responsible. Integrated and/or interoperable systems will be needed to avoid additional workload and errors due to loss of data or operational inaccuracies. *" If you have different suppliers managing that trial, then those different suppliers will most likely come up with their own systems [data management, randomisation, lab supplies, CTA management]. There will be several systems in a given trial […] that are not associated with each other at all"* (male, sponsor representative).

3.4. Main Challenges to the Digitalisation of Trials in Sub-Saharan Africa

Challenges for digital transformation in clinical trials are multifactorial. The main identified challenges were grouped under four categories and included (i) the usability and practicability of the technology; (ii) ethics and regulations; (iii) fear of paradigm shift; and (iv) site contextual factors (Figure 1).

3.4.1. Usability and Practicability of the Technology

One of the key challenges is the practical use of the technology and the lack of stable internet connectivity in some rural settings of sub-Saharan Africa. The maintenance of the digital tools could be complex too, as was mentioned by some investigators. *"When you collect the data electronically – when something goes wrong, how will it be taken care of? Maybe there are a few things that need to happen before we can do it"* (male, study coordinator).

Moreover, the initial set-up costs, the robustness, easiness of use, and the acceptability (especially for wearable devices) were mentioned as essential barriers to be address.

3.4.2. Paradigm Shift and Trial Data Quality

The ability of the investigators and participants to master novel technologies within their daily work was raised as a fundamental challenge. DDCTs will require a complete reorganisation and change of site processes, which means a substantial shift in clinical trial paradigm. *"It's true that we may be moving towards that [digitalisation], but in reality there will be a lot of efforts to bring people up to speed in order to achieve that. I dare to believe that COVID-19 or other similar events will not stop the usual process that we know."* (male, principal investigator).

For peer review only - http://bmjopen.bmj.com/site/about/guidelines.xhtml

Moreover, most investigators interviewed stressed that the change from conventional clinical trial operations to DDCTs could also jeopardize the quality of clinical trials at the beginning and pose a threat to data validity.

3.4.3. Ethics and Regulations

The fit of DDCTs within clinical trial regulations and their acceptance by ethics committees and regulatory authorities were identified as obstacles by investigators. Investigators indicated the discomfort resulting from the use of some technologies and the probable challenge for ethical clearance. This is reflected in the following statement regarding wearables devices: *"I think that ethically this will cause problems with the ethics committees because it's like an additional hygiene that we put the patients through- if you have to wear a "heart monitor" for 24 hours, plus two or three other things, it won't be easy"* (Male, principal investigator).

However, some investigators suggested mitigation strategies to address this challenge. These strategies included adequate communication, education of participants and investigators, and regulatory authority bodies on the risk-benefit assessment of the new tools.

3.4.4. Contextual Factors

Context specific factors will play an important role for the DDCTs implementation. These aspects are classified as research institutions, participants, and sociocultural related factors. Site experience in clinical research is key for DDCTs implementation. The main research focus in the visited African institutions was malaria, which could be treated in ambulatory care when uncomplicated without a long-term follow-up. This trend of therapeutic area specificities in our study was foreseen and may be viewed as a limitation for some aspects of DDCTs implementation. The collection of long-term and follow-up data were, however, touched in some interviews and concerns were brought up by both sponsors and investigators, as follows: *"Many of the things you need to measure are done in the clinic. I don't think we can take that approach with a malaria trial during the acute phase. In post-treatment, possibly, there might be a place for gathering adverse event information. Honestly, it's probably easier to have a healthcare worker or a nurse from the site visiting the community and gathering that information"* (female, sponsor representative).

Both sponsors and investigators also stressed the low literacy rate in some rural areas and the daily occupational activities as limitations for DDCTs application in sub-Saharan Africa, particularly for the eConsent. *"I don't think it's impossible, but it's going to be difficult, because we're in a context where this type of study involves the participant much more in the implementation of the trial itself, which assumes a certain level of education. It's going to be quite complicated at the moment"* (male, principal investigator).

For peer review only

For peer review only - http://bmjopen.bmj.com/site/about/guidelines.xhtml

Clinical trials' social acceptability and people's opinion and understanding were perceived as barriers to DDCTs implementation, as indicated by the notion of a respondent: *"The main challenge for us would be how to convince the patient"* (female, study coordinator).

Furthermore, interviewees also mentioned that the tools could create misunderstanding, trust disruption and dampen population participation in clinical trials. Adequate and timely communication was suggested to address this issue. *"I think that the socio-cultural context can also be a blocking factor -If we use these technologies – this can initially be a brake on the trust that exists between the researchers and the populations"* (male, study coordinator).

Both sponsors and investigators mentioned the possible disruption of the relationship between an investigator (as care provider) and a participant due to the use of new technologies: *"Physician-to-patient relationship is very important – we should not dehumanise trials as long as it is possible to do trials without putting investigators and volunteers at risk"* (male, principal investigator).

4. Discussion

We assessed the opportunities and challenges of DDCTs in sub-Saharan Africa from the perspectives of investigators, CROs, and sponsors. Against this background, we discussed the potential solutions for efficient DDCTs transformation in light of these perspectives. Clearly, DDCTs provide an opportunity for clinical research improvement in sub-Saharan Africa and elsewhere, despite certain challenges to be addressed, especially for research centres operating in rural or semi-rural settings. As for all technological transitions, the introduction of DDCTs requires technology mastering (training, technology suitability, and transfer and maintenance), and a comprehensive communication strategy with all parties involved. It also requires adaptation of digital tools to the study context and revision of both the current national and international regulations.

4.1. DDCTs Feasibility, Technology Mastering, and Context Adaptation

Technology feasibility and acceptability are important considerations in the transformation process from conventional clinical trials to DDCTs. Some aspects of the digitalisation such as the use of wearable devices for decentralised participants monitoring remain challenging. The implementation of DDCTs was set-back by the lack of infrastructure for remote participant engagement. Adoption of DDCTs during the COVID-19 pandemic was essential to ensure the continuity of ongoing trials on various medical indications, sponsor-CROs interactions (18) and initiation of COVID-19-related trials (19-21). A prerequisite was that during this transformation, DDCTs would not generate scientific and quality breaches (22). The new technologies will only be beneficial if their use is well mastered and clearly understood by all the actors', including research teams, sponsors, regulatory authorities, ethics committees, and participants.

For peer review only - http://bmjopen.bmj.com/site/about/guidelines.xhtml

Sponsors, sites and, where applicable, the supporting CROs should perform early and appropriate feasibility assessment, including site capacities prior to conduct DDCTs and suggest context adaptive improvements where necessary. User-friendly and multifunctional digital solutions (with possibility to measure several parameters, e.g. pulse, temperature, respiratory rate with one device) developed in sub-Saharan Africa and/or pilot testing are warranted. The multiplicity of the platforms used in digitalised trials for data acquisition and trial operations may become burdensome to the research teams. Hence, a careful selection of platforms to be used with the support of the investigators and considerations of the platforms integration and interoperability is required (23). Additionally, the availability or development of local technical expertise for the technology maintenance will be key for a long-term continuity of DDCTs. The commitment of sponsors to support sites for initial investment and the continuous training will be essential.

4.2. From Site-Centric to Participant-Centric Approaches

The shift from site-centric to participant-centric trials comes with a transfer of trial responsibilities to the participants. ePRO are an important component of this change and this needs substantial training of trial participants and investigators and a well-defined validation process. To mitigate acceptance issues and increase participants' buy-in, patient groups should be involved in the selection of patient-centred technologies whenever possible to enhance the success of DDCTs (24, 25). The technology and decentralisation processes should be integrated as a part of patients' follow-up with the support of their caregivers (health professionals) and in term reinforce the research teams and caregivers collaboration. Patient-centric technologies should be context adapted. Lastly, it is of paramount importance that the mentioned shift of responsibility to the patients will not waive or delay the medical care, the trial oversight by the principal investigator and the sponsor's responsibilities.

4.3. DDCTs for Clinical Trial Efficiency and Quality Improvement

The ultimate goal of DDCTs will be to improve trial quality and efficiency (processes, costs, and duration). For instance, although broadly in use, electronic data capture still bears the challenge of source data availability for verification and data reconciliation. This gap and the lack of clear guidelines for EDC tools being used as source data can also elicit ethics and regulatory authority's reluctance or hesitancy for the transition to DDCTs. The challenge of missing data in DDCTs due to poor quality of participant recordings or lack of interoperability should be addressed (26). In sub-Saharan Africa context, decentralised data collection with regular home visits by trial staff for participants monitoring or drug delivery combined with electronic data capture, could be an alternative solution. Recently, this approach was even proven to be more efficient than fully onsite data collection (27).

For peer review only - http://bmjopen.bmj.com/site/about/guidelines.xhtml

In addition, the lack of electronic health records in many countries particularly in resource-constrained settings makes participant recruitment and follow-up challenging due to the absence of screening database (28). Sponsor support to establishing interoperable and trustworthy electronic health records could be a cost-effective solution to reduce clinical trial duration and costs and at the same time result in health systems strengthening. Even considering a positive benefit-risk ratio of DDCTs, the initial investments and necessary logistics for DDCTs deployment may be costly, especially for small trials or those with a short follow-up period.

4.4. Data Privacy, Security, Sharing, and Ownership

Participants' privacy and data safety also remain concerns to be addressed for better deployment of DDCTs. As the use of internet-based solutions is often not risk-free (29, 30), one has to balance the remote data collection and prevent the collection of unnecessary individual confidential data, which could pose a threat to participant privacy. The collection of individual data could bear the possibility of participants' re-identification (31), especially for single site or small sample-size studies. Emerging technologies such as blockchains and tokenisation could enhance participant privacy (32, 33). However, several important questions remain as to whom has access, control, ownership of the trial data, and the storage place of the data. These issues were partially addressed by the current ICH E6 R2 requirements empowering the sites and investigators (12). DDCTs should not waive these rights from the sites. Trustful, clear data storage and sharing policy should be adopted in countries where these are missing, in addition to the fulfilment of other ICH requirements.

4.5. Ethical and Regulatory Considerations to DDCTs

The digital transformation was delayed due to the lack of clarity of clinical trial regulations (2). The subsequent revisions 2 and 3 of ICH E6 guideline underline the need for novel technologies used and revision 3 is expected to reinforce and clarify this transformation (34). This will potentially alleviate the burden of trial processes for investigators, participants, and sponsors (12, 35). The COVID-19 pandemic has also fostered the adoption of decentralised solutions by regulatory authorities for site inspections during the pandemic (36). Hence, DDCTs' were addressed by the US Food and Drug Administration (FDA) (37) and the EMA and their implementation was encouraged. These guidelines triggered pharmaceutical sponsors' interest in DDCTs, which was previously dominated by investigator-initiated trials (38). However, in sub-Saharan Africa, in the absence of a fully functioning African Medicine Agency (AMA), most of national regulatory authorities have thus far not revised their guidelines contributing to the gap in DDCTs uptake. There is a need for a well-established AMA (39) to adequately oversee DDCTs implementation. Investigators and sponsors could play an

For peer review only

For peer review only - http://bmjopen.bmj.com/site/about/guidelines.xhtml

important role for regulatory and ethics bodies by providing them updated information and training on available digital and decentralised solutions (40). The digital tools to be used should be specified in the protocol to reduce the potential breaches of clinical trials' core principles, particularly the participants' protection (35, 41).

4.6. Community Engagement and Communication

The importance of community engagement in clinical research and health interventions is well established and several frameworks have been used for its assessment (42, 43). DDCTs offered the opportunity for better community engagement in clinical trials through several means, such as crowdsourcing (44), virtual advertisement of trials and both diversity of study participants and geographic representativity (45). These points can only be accomplished when clinical trial processes are well conveyed to the parties (i.e., participants and community leaders) before trial initiation and during the informed consent procedure. Continuous involvement of community leaders and local health authorities in the research process is likely to minimise misinformation, which is crucial to ensure future research activities (46). In this light, priority should be given to transparent communication, the interaction between investigators and participants, and the participants' ability to attend and meet the study team anytime during trial participation. In sub-Saharan Africa, the conventional recruitment strategy with direct engagement with the community is also proven to be efficient with a low rate of loss to follow-up (47); this should not be waved during digitalisation and decentralisation. A hybrid recruitment model with media and online advertisement of trials on social media (48), combined with the on-site community engagement, warrants further consideration.

For peer review only

4.7. Potential Global Health Implications of DDCTs

The potential benefits and challenges of DDCTs adoption in sub-Saharan Africa and elsewhere should be explored without further delay (23). In addition, by allowing real-world data collection through methods such as the tokenisation and AI, trial participants could be followed-up for longer periods after completing a trial. Such data could serve for monitoring delayed and rare adverse events and identification of potentially new indications.

Moreover, the establishment of electronic health records and their use in clinical trials for participant selection and folllow-up could play an important role in sub-Saharan Africa, thus contribute to improve chronic diseases research, including cancer research (49). Hence, the digital transformation could contribute to strenghten capacities in sub-Saharan Africa and broaden the spectrum of clinical research by addressing both communicable and non-communicable diseases with reduced risk of long term loss of follow-up.

4.8. Strengths and Limitations

For peer review only - http://bmjopen.bmj.com/site/about/guidelines.xhtml

This study aimed at filling a current knowledge gap pertaining to DDCTs in sub-Saharan Africa. As the data were collected during the COVID-19 pandemic, the thriving of DDCTs in this period allowed the participants to better appraise the opportunities and challenges of DDCTs in their context. The study was conducted in experienced, well-equipped clinical trial sites, with a diversified research portfolio. However, the interviewed professionals and visited sites did not yet have broad experience with some digital solutions use in clinical trials, which could have limited their perception of these technologies.

5. Conclusion

The digital transformation in clinical research in sub-Saharan Africa has evolved in the post-COVID-19 era. DDCTs favoured the adaptation and continuity of clinical trials during the pandemic and enhanced the tasks shift from sponsor to investigators and from investigators to patients. If appropriately implemented, DDCTs could serve as an important mean to improve clinical trial efficiency despite the multifactorial challenges to be addressed. This ineluctable transformation could also be detrimental without proper planning, early involvement of users, and adequate site capabilities assessment for DDCTs implementation. A stepwise transformation, the commitment of ethics, investigators, sponsors, and regulatory authorities, and the buy-in of the communities are essential for a successful DDCTs adoption. There is a need for substantial technological and logistic investment, training, communication, and community engagement. The ongoing revision 3 of the ICH E6 guideline offers a unique opportunity to pursue the conversation for better implementation of DDCTs. As DDCTs are patient centric, solid, and contextualised considerations with regard to good participant-investigator relationships and mutual trust should inform the digital transformation.

Acknowledgements

We express our deep gratitude to all the interviewees and sites (Malaria Research and Training Centre- Parasitology in Mali, the Unité de Recherche Clinique de Nanoro in Burkina Faso, the University Hospital Basel in Switzerland) and the “African Contribution to Global Health” teams for their support. We are grateful to our scientific advisory committee members for their extended support.

Authors’ contributions

EN contributed to the study design, data collection, data analysis and wrote the first draft. CB contributed to the design, data analysis, the manuscript writing and review. HS contributed to data analysis and manuscript review. PE provided methodological guidance and contributed to the manuscript writing and review. ER, AS and JU contributed to the manuscript writing and review.

For peer review only - http://bmjopen.bmj.com/site/about/guidelines.xhtml

Ethical Clearance

The study was approved by the Comité d'Éthique pour la Recherche en Santé (CERS) in Burkina Faso and the Comité National d'Éthique in Mali. Additionally, the study was approved by the research commission of the Swiss Tropical and Public Health Institute (Swiss TPH), and an exemption was obtained from the Ethics Committee of Northwestern and Central Switzerland (EKZN). All interview records were kept on a server with secured access to maintain confidentiality.

Funding

This study was funded by the Swiss National Science Foundation (SNSF) within the project "African Contribution to Global Health" (grant number CRSII5_183577).

Competing interests

None declared

Data availability statement

The data are available and could be shared upon reasonable request.

For peer review only

References

1. Manyazewal T, Woldeamanuel Y, Blumberg HM, Fekadu A, Marconi VC. The potential use of digital health technologies in the African context: a systematic review of evidence from Ethiopia. NPJ Digit Med. 2021;4(1):125.

2. Banks MA. Core Concept: In the wake of COVID-19, decentralized clinical trials move to center stage. Proc Natl Acad Sci U S A. 2021;118(47).

3. Rogers A, De Paoli G, Subbarayan S, Copland R, Harwood K, Coyle J, et al. A systematic review of methods used to conduct decentralised clinical trials. Br J Clin Pharmacol. 2022;88(6):2843-62.

4. Grignolo A. The Clinical Trials Transformation Initiative (CTTI). Ann Ist Super Sanita. 2011;47(1):14-8.

5. Inan OT, Tenaerts P, Prindiville SA, Reynolds HR, Dizon DS, Cooper-Arnold K, et al. Digitizing clinical trials. NPJ Digit Med. 2020;3(1):101.

6. Orri M, Lipset CH, Jacobs BP, Costello AJ, Cummings SR. Web-based trial to evaluate the efficacy and safety of tolterodine ER 4 mg in participants with overactive bladder: REMOTE trial. Contemp Clin Trials. 2014;38(2):190-7.

7. Goldsack JC, Izmailova ES, Menetski JP, Hoffmann SC, Groenen PMA, Wagner JA. Remote digital monitoring in clinical trials in the time of COVID-19. Nat Rev Drug Discov. 2020;19(6):378-9.

8. Blatch-Jones A, Nuttall J, Bull A, Worswick L, Mullee M, Peveler R, et al. Using digital tools in the recruitment and retention in randomised controlled trials: survey of UK Clinical Trial Units and a qualitative study. Trials. 2020;21(1):304.

9. Nissen J. Do digital clinical trials offer hope for patients? Popul Health Manag. 2020;23(4):273-5.

10. van Dam J, Omondi Onyango K, Midamba B, Groosman N, Hooper N, Spector J, et al. Open-source mobile digital platform for clinical trial data collection in low-resource settings. BMJ Innov. 2017;3(1):26-31.

11. Peyser ND, Marcus GM, Beatty AL, Olgin JE, Pletcher MJ. Digital platforms for clinical trials: The Eureka experience. Contemp Clin Trials. 2022;115:106710.

12. ICH. International council for harmonisation of technical requirements for pharmaceuticals for human use (ICH) 2022 [Available from: https://ichgcp.net/. Accessed 12.02.2023]

13. Sato T, Ishimaru H, Takata T, Sasaki H, Shikano M. Application of internet of medical/health things to decentralized clinical trials: development status and regulatory considerations. front Med (Lausanne). 2022;9:903188.

14. EMA. Reflection paper on expectations for electronic source data and data transcribed to electronic data collection tools in clinical trials. 2010.

15. Djimde A, Ranque S, Thera MA. Building a center of excellence in biomedical research in an unfavorable environment: the Malaria Research and Training Center in Mali. Med Sante Trop. 2019;29(4):343-7.

For peer review only

For peer review only - http://bmjopen.bmj.com/site/about/guidelines.xhtml

16. van Zyl-Hermann D, Hammel T, Burri C, Chenal J, Fink G, Konou AA, et al. Examining African contributions to global health: Reflections on knowledge circulation and innovation. Glob Public Health. 2022:1-16.

17. Phillippi J, Lauderdale J. A guide to field notes for qualitative research: context and conversation. Qual Health Res. 2018;28(3):381-8.

18. Xue JZ, Smietana K, Poda P, Webster K, Yang G, Agrawal G. Clinical trial recovery from COVID-19 disruption. Nat Rev Drug Discov. 2020;19(10):662-3.

19. GlobalData Healthcare. The dynamics of clinical research in Africa: 2016-2021 2022 [Available from: https://www.clinicaltrialsarena.com/comment/clinical-research-africa-2016-21/. Accessed 20.12.2022]

20. Afroz MA, Schwarber G, Bhuiyan MAN. Risk-based centralized data monitoring of clinical trials at the time of COVID-19 pandemic. Contemp Clin Trials. 2021;104:106368.

21. Yamada O, Chiu SW, Takata M, Abe M, Shoji M, Kyotani E, et al. Clinical trial monitoring effectiveness: Remote risk-based monitoring versus on-site monitoring with 100% source data verification. Clin Trials. 2021;18(2):158-67.

22. Thakur S, Lahiry S. Digital clinical trial: A new norm in clinical research. Perspect Clin Res. 2021;12(4):184-8.

23. Clinical research in resource-limited settings. A consensus by a CIOMS Working Group., (2021).

24. Dockendorf MF, Hansen BJ, Bateman KP, Moyer M, Shah JK, Shipley LA. Digitally Enabled, Patient-Centric Clinical Trials: Shifting the Drug Development Paradigm. Clin Transl Sci. 2021;14(2):445-59.

25. Mowlem FD, Tenaerts P, Gwaltney C, Oakley-Girvan I. Regulatory Acceptance of Patient-Reported Outcome (PRO) Data from Bring-Your-Own-Device (BYOD) Solutions to Support Medical Product Labeling Claims Let's Share the Success Stories to Move the Industry Forward. Therapeutic Innovation & Regulatory Science. 2022;56(4):531-5.

26. Di J, Demanuele C, Kettermann A, Karahanoglu FI, Cappelleri JC, Potter A, et al. Considerations to address missing data when deriving clinical trial endpoints from digital health technologies. Contemp Clin Trials. 2022;113:106661.

27. Curtis A, Qu Y. Impact of Using A Mixed Data Collection Modality on Statistical Inferences in Decentralized Clinical Trials. Ther Innov Regul Sci. 2022;56(5):744-52.

28. Laaksonen N, Bengtstrom M, Axelin A, Blomster J, Scheinin M, Huupponen R. Success and failure factors of patient recruitment for industry-sponsored clinical trials and the role of the electronic health records-a qualitative interview study in the Nordic countries. Trials. 2022;23(1):385.

29. De Brouwer W, Patel CJ, Manrai AK, Rodriguez-Chavez IR, Shah NR. Empowering clinical research in a decentralized world. NPJ Digit Med. 2021;4(1):102.

30. Munos B, Baker PC, Bot BM, Crouthamel M, de Vries G, Ferguson I, et al. Mobile health: the power of wearables, sensors, and apps to transform clinical trials. Ann N Y Acad Sci. 2016;1375(1):3-18.

For peer review only - http://bmjopen.bmj.com/site/about/guidelines.xhtml

31. Maritsch F, Cil I, McKinnon C, Potash J, Baumgartner N, Philippon V, et al. Data privacy protection in scientific publications: process implementation at a pharmaceutical company. BMC Med Ethics. 2022;23(1):65.

32. Zhuang Y, Zhang L, Gao X, Shae ZY, Tsai JJP, Li P, et al. Re-engineering a Clinical Trial Management System Using Blockchain Technology: System Design, Development, and Case Studies. J Med Internet Res. 2022;24(6):e36774.

33. Huh KY, Moon SJ, Jeong SU, Kim MJ, Yang W, Jeong M, et al. Evaluation of a blockchain-based dynamic consent platform (METORY) in a decentralized and multicenter clinical trial using virtual drugs. Clin Transl Sci. 2022;15(5):1257-68.

34. Stansbury N, Barnes B, Adams A, Berlien R, Branco D, Brown D, et al. Risk-Based Monitoring in Clinical Trials: Increased Adoption Throughout 2020. Ther Innov Regul Sci. 2022;56(3):415-22.

35. de Jong AJ, van Rijssel TI, Zuidgeest MGP, van Thiel G, Askin S, Fons-Martinez J, et al. Opportunities and Challenges for Decentralized Clinical Trials: European Regulators' Perspective. Clin Pharmacol Ther. 2022;112(2):344-52.

36. Demasi M. FDA oversight of clinical trials is "grossly inadequate," say experts. BMJ (Clinical research ed). 2022;379:o2628.

37. FDA. Digital health innovation plan. In: Administration MUSFaD, editor.: Silver Spring; 2020.

38. Deplanque D, Cviklinski S, Bardou M, Ader F, Blanchard H, Barthelemy P, et al. Health crisis: What opportunities for clinical drug research? Therapie. 2022;77(1):59-67.

39. Solarin O, Mohammed SI, Ndlovu N, Vanderpuye V, Olaiya V. Partnerships and Collaborations: The Right Alliances for Clinical Trials in Africa. JCO Glob Oncol. 2020;6:954-8.

40. Marquis-Gravel G, Roe MT, Turakhia MP, Boden W, Temple R, Sharma A, et al. Technology-Enabled Clinical Trials: Transforming Medical Evidence Generation. Circulation. 2019;140(17):1426-36.

41. van Rijssel TI, de Jong AJ, Santa-Ana-Tellez Y, Boeckhout M, Zuidgeest MGP, van Thiel G, et al. Ethics review of decentralized clinical trials (DCTs): Results of a mock ethics review. Drug Discov Today. 2022;27(10):103326.

42. Sofolahan-Oladeinde Y, Newhouse RP, Lavallee DC, Huang JC, Mullins CD. Early assessment of the 10-step patient engagement framework for patient-centred outcomes research studies: the first three steps. Fam Pract. 2017;34(3):272-7.

43. Andrasik MP, Broder GB, Wallace SE, Chaturvedi R, Michael NL, Bock S, et al. Increasing Black, Indigenous and People of Color participation in clinical trials through community engagement and recruitment goal establishment. PLoS One. 2021;16(10):e0258858.

44. Day S, Mathews A, Blumberg M, Vu T, Mason H, Rennie S, et al. Expanding community engagement in HIV clinical trials: a pilot study using crowdsourcing. AIDS. 2020;34(8):1195-204.

45. Tan RKJ, Wu D, Day S, Zhao Y, Larson HJ, Sylvia S, et al. Digital approaches to enhancing community engagement in clinical trials. NPJ Digit Med. 2022;5(1):37.

For peer review only

For peer review only - http://bmjopen.bmj.com/site/about/guidelines.xhtml

664 46. Adebesin F, Mwalugha R. The Mediating Role of Organizational Reputation and Trust in the Intention to Use Wearable Health Devices: Cross-Country Study. JMIR Mhealth Uhealth. 2020;8(6):e16721.

667 47. Idoko OT, Owolabi OA, Odutola AA, Ogundare O, Worwui A, Saidu Y, et al. Lessons in participant retention in the course of a randomized controlled clinical trial. BMC Res Notes. 2014;7:706.

670 48. Gunturu KS, Dizon DS, Johnson J, Mercurio AM, Mason G, Sparks DB, et al. Clinical trials in the era of digital engagement: a SWOG call to action. JCO Clin Cancer Inform. 2020;4:254-8.

673 49. Odekunle FF, Odekunle RO, Shankar S. Why sub-Saharan Africa lags in electronic health record adoption and possible strategies to increase its adoption in this region. Int J Health Sci (Qassim). 2017;11(4):59-64.

For peer review only

For peer review only - http://bmjopen.bmj.com/site/about/guidelines.xhtml

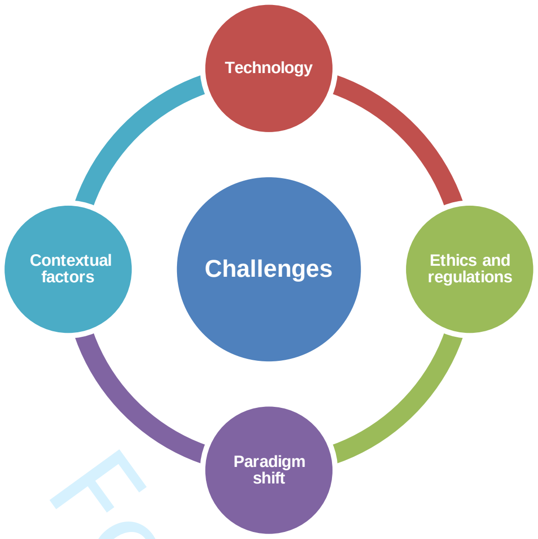

Figure 1: Challenges for decentralised and digitalised clinical trials in sub-Saharan Africa

For peer review only

Standards for Reporting Qualitative Research (SRQR)*

http://www.equator-network.org/reporting-guidelines/srqr/

Title and abstract

	Page/line no(s).
**Title** - Concise description of the nature and topic of the study Identifying the study as qualitative or indicating the approach (e.g., ethnography, grounded theory) or data collection methods (e.g., interview, focus group) is recommended	Page 1, line 1-2
**Abstract** - Summary of key elements of the study using the abstract format of the intended publication; typically includes background, purpose, methods, results, and conclusions	Page 3; line 30-59

Introduction

**Problem formulation** - Description and significance of the problem/phenomenon studied; review of relevant theory and empirical work; problem statement	Page 4-5; lines 61-103
**Purpose or research question** - Purpose of the study and specific objectives or questions	Page 4-5; lines 61-103

Methods

**Qualitative approach and research paradigm** - Qualitative approach (e.g., ethnography, grounded theory, case study, phenomenology, narrative research) and guiding theory if appropriate; identifying the research paradigm (e.g., postpositivist, constructivist/ interpretivist) is also recommended; rationale**	Pages 5-6; lines 119-151
**Researcher characteristics and reflexivity** - Researchers' characteristics that may influence the research, including personal attributes, qualifications/experience, relationship with participants, assumptions, and/or presuppositions; potential or actual interaction between researchers' characteristics and the research questions, approach, methods, results, and/or transferability	Pages 4-5; lines 119-151
**Context** - Setting/site and salient contextual factors; rationale**	Pages 5; lines 107-117
**Sampling strategy** - How and why research participants, documents, or events were selected; criteria for deciding when no further sampling was necessary (e.g., sampling saturation); rationale**	Pages 5; lines 119-126
**Ethical issues pertaining to human subjects** - Documentation of approval by an appropriate ethics review board and participant consent, or explanation for lack thereof; other confidentiality and data security issues	Pages 16; lines 525-531
**Data collection methods** - Types of data collected; details of data collection procedures including (as appropriate) start and stop dates of data collection and analysis, iterative process, triangulation of sources/methods, and modification of procedures in response to evolving study findings; rationale**	Pages 6; lines 153-163

For peer review only - http://bmjopen.bmj.com/site/about/guidelines.xhtml

**Data collection instruments and technologies** - Description of instruments (e.g., interview guides, questionnaires) and devices (e.g., audio recorders) used for data collection; if/how the instrument(s) changed over the course of the study	Pages 6; lines 117-148
**Units of study** - Number and relevant characteristics of participants, documents, or events included in the study; level of participation (could be reported in results)	Pages 6; lines 171-179
**Data processing** - Methods for processing data prior to and during analysis, including transcription, data entry, data management and security, verification of data integrity, data coding, and anonymization/de-identification of excerpts	Pages 6; lines 153-163
**Data analysis** - Process by which inferences, themes, etc., were identified and developed, including the researchers involved in data analysis; usually references a specific paradigm or approach; rationale**	Pages 4-5; lines 153-163
**Techniques to enhance trustworthiness** - Techniques to enhance trustworthiness and credibility of data analysis (e.g., member checking, audit trail, triangulation); rationale**	Pages 6; lines 150-160

Results/findings

**Synthesis and interpretation** - Main findings (e.g., interpretations, inferences, and themes); might include development of a theory or model, or integration with prior research or theory	Pages 7-12; lines 171-351
**Links to empirical data** - Evidence (e.g., quotes, field notes, text excerpts, photographs) to substantiate analytic findings	Pages 7-12; lines 171-351

Discussion

**Integration with prior work, implications, transferability, and contribution(s) to the field -** Short summary of main findings; explanation of how findings and conclusions connect to, support, elaborate on, or challenge conclusions of earlier scholarship; discussion of scope of application/generalizability; identification of unique contribution(s) to scholarship in a discipline or field	Pages 1-15; Lines 353-493
**Limitations** - Trustworthiness and limitations of findings	Page 15; Lines 486-493

Other

**Conflicts of interest** - Potential sources of influence or perceived influence on study conduct and conclusions; how these were managed	Page 16; line 537-538
**Funding** - Sources of funding and other support; role of funders in data collection, interpretation, and reporting	Page 16; line 533-535

For peer review only - http://bmjopen.bmj.com/site/about/guidelines.xhtml

*The authors created the SRQR by searching the literature to identify guidelines, reporting standards, and critical appraisal criteria for qualitative research; reviewing the reference lists of retrieved sources; and contacting experts to gain feedback. The SRQR aims to improve the transparency of all aspects of qualitative research by providing clear standards for reporting qualitative research.

**The rationale should briefly discuss the justification for choosing that theory, approach, method, or technique rather than other options available, the assumptions and limitations implicit in those choices, and how those choices influence study conclusions and transferability. As appropriate, the rationale for several items might be discussed together.

Reference:
O'Brien BC, Harris IB, Beckman TJ, Reed DA, Cook DA. **Standards for reporting qualitative research: a synthesis of recommendations.** *Academic Medicine*, Vol. 89, No. 9 / Sept 2014 DOI: 10.1097/ACM.0000000000000388

For peer review only

BMJ Open

Opportunities and challenges for decentralised clinical trials in sub-Saharan Africa: a qualitative study

Journal:	*BMJ Open*
Manuscript ID	bmjopen-2023-075903.R1
Article Type:	Original research
Date Submitted by the Author:	04-Aug-2023
Complete List of Authors:	Nebie, Eric; Centre de Recherche en Sante de Nouna, ; Swiss TPH, Medicines Implementation Research Unit<br>Sawadogo, Hélène N.; Centre de Recherche en Santé de Nouna<br>van Eeuwijk, Peter; Swiss Tropical and Public Health Institute; University of Basel<br>Signorell, Aita ; Swiss Tropical and Public Health Institute; University of Basel<br>Reus, Elisabeth; Swiss Tropical and Public Health Institute; University of Basel<br>Utzinger, Juerg; Swiss Tropical and Public Health Institute; University of Basel<br>Burri, Christian; Swiss Tropical and Public Health Institute; University of Basel
**Primary Subject Heading**:	Public health
Secondary Subject Heading:	Qualitative research
Keywords:	Clinical Trial, QUALITATIVE RESEARCH, PUBLIC HEALTH

SCHOLARONE™
Manuscripts

I, the Submitting Author has the right to grant and does grant on behalf of all authors of the Work (as defined in the below author licence), an exclusive licence and/or a non-exclusive licence for contributions from authors who are: i) UK Crown employees; ii) where BMJ has agreed a CC-BY licence shall apply, and/or iii) in accordance with the terms applicable for US Federal Government officers or employees acting as part of their official duties; on a worldwide, perpetual, irrevocable, royalty-free basis to BMJ Publishing Group Ltd ("BMJ") its licensees and where the relevant Journal is co-owned by BMJ to the co-owners of the Journal, to publish the Work in this journal and any other BMJ products and to exploit all rights, as set out in our licence.

The Submitting Author accepts and understands that any supply made under these terms is made by BMJ to the Submitting Author unless you are acting as an employee on behalf of your employer or a postgraduate student of an affiliated institution which is paying any applicable article publishing charge ("APC") for Open Access articles. Where the Submitting Author wishes to make the Work available on an Open Access basis (and intends to pay the relevant APC), the terms of reuse of such Open Access shall be governed by a Creative Commons licence – details of these licences and which Creative Commons licence will apply to this Work are set out in our licence referred to above.

Other than as permitted in any relevant BMJ Author's Self Archiving Policies, I confirm this Work has not been accepted for publication elsewhere, is not being considered for publication elsewhere and does not duplicate material already published. I confirm all authors consent to publication of this Work and authorise the granting of this licence.

For peer review only

Opportunities and challenges for decentralised clinical trials in sub-Saharan Africa: a qualitative study

Eric I. Nebié*[1,2,3], Hélène N. Sawadogo[3], Peter van Eeuwijk[1,2,4], Aita Signorell[1,2], Elisabeth Reus[1,2], Juerg Utzinger[1,2] and Christian Burri[1,2]

[1]Swiss Tropical and Public Health Institute, Kreuzstrasse 2, CH-4123 Allschwil, Switzerland
[2]University of Basel, Petersplatz 1, CH-4003 Basel, Switzerland
[3]Centre de Recherche en Santé de Nouna, Street Namory Keita, P.O. Box 02, Nouna, Burkina Faso
[4]Institute of Social Anthropology, University of Basel, Münsterplatz 19, CH-4051 Basel, Switzerland

*Corresponding author:
Eric Ipyn Nebié
Centre de Recherche en Santé de Nouna, Street Namory Keita, P.O. Box 02, Nouna, Burkina Faso
Swiss Tropical and Public Health Institute, Kreuzstrasse 2, CH-4123 Allschwil, Switzerland
Email: neric83@yahoo.fr

For peer review only

Abstract

Introduction

Digital health has gained traction in research and development, and clinical decision support systems. The COVID-19 pandemic accelerated the adoption of decentralised clinical trials (DCTs) as a mitigation and efficiency improvement strategy. We assessed the opportunities and challenges of a digital transformation in clinical research in sub-Saharan Africa from different stakeholders' perspectives.

Methods

A qualitative study, including 40 in-depth semi-structured interviews, was conducted with investigators of three leading research institutions in sub-Saharan Africa and Switzerland, contract research organisations and sponsors managing clinical trials in sub-Saharan Africa. A thematic approach was used for the analysis.

Results

Interviewees perceived DCTs as an opportunity for trial efficiency improvement, quality improvement and reducing the burden of people participating in clinical trials. However, to gain and maintain an optimal quality of clinical trials, a transition period is necessary to tackle contextual challenges before DCTs are being implemented. The main challenges are categorised into four themes: (i) usability and practicability of the technology; (ii) paradigm shift and trial data quality; (iii) ethical and regulatory hurdles; and (iv) contextual factors (site-specific research environment and sociocultural aspects).

Conclusion

The transformation from a site to a patient centric model with an increased responsibility of participants should be context adapted. The transformation requires substantial investment, training of the various stakeholders and an efficient communication. Additionally, commitment of sponsors, investigators, ethics, and regulatory authorities and the buy-in of the communities are essential for this change.

Keywords: Clinical research; clinical trials; decentralised clinical trials; digital health; qualitative research sub-Saharan Africa

For peer review only

Strengths and limitations

- This study provides insights into opportunities and challenges of digitalisation and decentralisation of clinical trials in sub-Saharan Africa from the perspectives and experience of investigators, contract research organisations and sponsors.
- The study was conducted in well-established research institutions, characterised by diversified research portfolios pertaining to several therapeutic areas and research context and experienced investigators.
- The conduct of the study after the onset of the COVID-19 pandemic and the increased adoption of alternative digital and decentralised solutions in research was an asset.
- This study did not include opinions of study participants who might have different perceptions than clinical trial investigators, contract research organisation and sponsors.

For peer review only - http://bmjopen.bmj.com/site/about/guidelines.xhtml

1. Introduction

Digital health technologies (DHT) are prominent innovations in global health as they hold promise to improve routine health care and foster the implementation of new health interventions (1). Decentralised clinical trials (DCTs) refers to the remote conduct of trial related activities out of the research site/clinic (2). The implementation of DCT focuses on the use of DHT (3) and include all remote engagement with trial participants even without the DHT. The concept of DCTs is participant-centred (2). DCTs are being developed as a promising solution for future clinical research (4) to address the productivity crisis in drug research and development with continuously increasing costs, duration, burden and complexity of clinical trials (5, 6). The first documented exclusive DCT (i.e., without any participant visit to the clinic), was the "REMOTE trial", which provided the basis for future DCTs (7). Prior to the COVID-19 pandemic, electronic data capture (EDC), electronic health records and telemedicine were the main digitalised areas in clinical trials. Recently, the use of wearable devices for electronic patient reported outcome (ePRO) and electronic clinical outcome assessment (eCOA), as well as artificial intelligence (AI), were gaining interest (8). The COVID-19 pandemic forcedly shed light on the opportunities for fast-track medical product development, while ensuring continuity of trials under the pandemic's stringent restrictions (9). DCTs have proven to be feasible and to have potential to improve participant recruitment, retention and representability in clinical trials (10, 11), data collection (12) and efficiency (13). However, in a recent systematic review covering studies conducted in Asia, Australia, Europe and North America, there was little consensual added value of digitalisation in clinical research (4).

Before the COVID-19 pandemic, DCTs were not fully covered by ethics and regulatory guidelines. Guidance on electronic records and electronic signatures were first included into the US Code of Federal Regulations Title 21, Part 11 (21 CFR Part 11). The first version and the revision 2 of the International Conference on Harmonization (ICH) Guideline E6 Good Clinical Practices (GCP) of 1996 (14) mentioned the use of digital and decentralised solutions in a few instances; yet, in a rather superficial way. Hence, the adoption of DCTs was challenging. By February 2022, only two countries (Denmark and Switzerland) had digital clinical trials included in their guidelines (15). In-depth deliberation of the topic was initiated in 2010 with a European Medicines Agency (EMA) position paper covering electronic source data and data transcribed to electronic data collection tools and was further detailed in 2023 in the Guideline on computerised systems and electronic data in clinical trials (16). The upcoming revision 3 of the ICH GCP and World Health Organization (WHO) guidance for best practices for clinical trials under public consultation, provides more details and encourages the use of DCTs to improve clinical trials efficiency (14, 17).

In sub-Saharan Africa, the transition to DCTs is at an early stage. With the outset and rapid spread of SARS-CoV-2, DCTs were expeditiously initiated in some research institutions

For peer review only - http://bmjopen.bmj.com/site/about/guidelines.xhtml

in sub-Saharan Africa to allow continuity of ongoing clinical trials despite the unfolding pandemic. The current study aimed to address the opportunities and challenges for the transition to DCTs, placing particular emphasis on sub-Saharan Africa.

2. Methods

2.1. Study Settings

Data were collected in two leading clinical research institutions in sub-Saharan Africa; the Malaria Research and Training Centre (MRTC) in Mali and the Clinical Research Unit of Nanoro (URCN) in Burkina Faso. Additionally, data were obtained from four departments of the University Hospital Basel (USB) in Switzerland, which are conducting clinical trials. The USB is also involved in clinical trials conducted in various settings in sub-Saharan Africa.

The two African research institutions have a long-standing clinical research experience with various sponsors from academia, product development partnerships (PDPs) and the pharmaceutical industry. These centres have strong capacities in terms of infrastructures, equipment and human resources and comply with international standards (18). The two research institutions' catchment areas include both rural and semi-urban settings; they operate in research clinics, hospitals and communities.

2.2. Study Design

We pursued a qualitative study, embedded in a sequential exploratory mixed method design in which a qualitative study component informed the development of the quantitative component (19). The qualitative component aimed to identify and explore the efficiency drivers of clinical development from various stakeholders' perspectives. Additionally, a 6-week work shadowing was performed in the headquarters of the institutions and the clinics/hospitals where the research was conducted. Observations and document reviews were performed to better understand study operations processes and the research context in each of the three purposively selected research institutions (MRTC, CRUN and USB). The work shadowing was delayed from May to December 2020 due to the lockdowns imposed by the COVID-19 pandemic. The presented work focused on the opportunities and challenges for digitalisation and decentralisation in clinical research assessed through the qualitative interviews. This topic was triggered by the escalating use of DCTs, particularly during and after the COVID-19 pandemic.

2.3. Interviews

The in-depth semi-structured interviews were conducted with clinical researchers (principal investigators and study coordinators), sponsors (project managers, project leaders,

For peer review only - http://bmjopen.bmj.com/site/about/guidelines.xhtml

clinical development staff, quality and regulatory affairs responsibles, and medical officers) and contract research organisations (CROs) managers who have been involved in at least one clinical trial in the past 3 years in sub-Saharan Africa. Three different interview guidelines were used for the investigators, sponsors and CROs. The main themes covered during the interviews were the interviewees' experience with DCTs before and during the COVID-19 pandemic, their perceptions of DCTs, potential benefits for sponsors and sites and, the perceived challenges for DCTs implementation.

A scientific advisory committee, comprising representatives from each sponsor type and a sub-Saharan Africa research institution leader (one from pharmaceutical industry, for profit; one from a biotech company, for profit; one from a PDP, not for profit; and one from academia), reviewed all the tools (interview guides, document review guide and work shadowing tools). Three members were working in Swiss-based companies with clinical research experience in sub-Saharan Africa and one in a research institution in Burkina Faso. The committee members provided inputs based on their expertise in clinical research. They were not interviewed and did not have any direct relation with the visited sites. The study was neither commissioned nor influenced by any type of sponsor.

2.4. Data Collection and Analysis

The work shadowing took place from December 2020 to October 2021. Interviews were either performed face-to-face (during the 6-week stay in the sites) or virtually using a Zoom license maintained by the Swiss Tropical and Public Health Institute (Swiss TPH; Allschwil, Switzerland). The interviews were conducted by the first author either in English or in French, according to the interviewees' preference. The interviews lasted between 45 and 90 min, depending on the interviewees' experience. The interviews were recorded, transcribed, translated, coded and analysed using MAXQDA version 20 (VERBI Software 2021; Berlin, Germany). A deductive and inductive thematic analysis was pursued. Two researchers (EIN and HNS) independently performed the coding after an extended reading of the transcripts. Subcodes and codes were generated and grouped into themes. Some preconceived themes were identified from the existing literature as the deductive analysis. Data were inductively analysed and new themes identified. Data were triangulated with the context understanding gained from the work shadowing. Contextual information was recorded and summarised in a "field journal" (20).

2.5. Researchers' Characteristic, Reflexivity and Trustworthiness

The study was conducted by researchers of Swiss TPH and the Centre de Recherche en Santé de Nouna (CRSN) in Burkina Faso. All involved researchers have extensive experience in the conduct of clinical studies. In addition, all of them had received specific trainings in

For peer review only - http://bmjopen.bmj.com/site/about/guidelines.xhtml

qualitative research and made previous contributions to mixed method studies about the quality and efficiency of clinical studies, and immunization mainly in resource-constrained settings(21-24). The researchers did not have working experience with the sites where the study was conducted. EIN was introduced to the teams by senior staff at Swiss TPH through the site leaders. Participants' were given the opportunity to ask for clarifications after reading the study information sheet. The research team was aware that a commissioned study by the sponsor could induce significant bias. Hence, it was mentioned clearly to participants that the study is conducted independently and not commissioned by any sponsor. Participants felt comfortable to share their views regarding the topic. EIN and HNS are based and worked in sub-Saharan Africa and have a deep understanding of the research context. The credibility was ensured by selecting participants with different roles and responsibilities in clinical trials. As the data were subsequently collected in different research sites, it was safeguarded that the interviews were not affected by the insights gained in the different contexts and the interviews were similar and comparable. The findings were discussed within the team to ensure that the results are correctly interpreted within the context. The current findings could be transferred in research environments sharing several commonalities in sub-Saharan Africa.

2.6. Patient and Public Involvement Statement

We did not include patients in the design, conduct, reporting and dissemination plan of the study. Instead, the study was conducted with investigators, CROs managers and clinical research sponsors staff.

3. Results

Overall, 40 in-depth semi-structured interviews were conducted. Previous experience of DCTs before and after the onset of the COVID-19 pandemic, perceived context-specific DCTs opportunities and challenges, and potential solutions were discussed during the interviews. The interviewees' characteristics are summarised in Table 1.

For peer review only

For peer review only - http://bmjopen.bmj.com/site/about/guidelines.xhtml

Table 1: Interviewees' characteristics

Participants' profiles		Number
**Gender**		
	**Male**	29
	**Female**	11
	**Total**	40
**Country of residence**		
	**Burkina Faso**	8
	**Germany**	2
	**Kenya**	1
	**Mali**	9
	**Senegal**	1
	**South Africa**	2
	**Switzerland**	16*
	**USA**	1
	**Total**	40
**Main role**		
	**CRO managers**	4
	**Principal investigators**	12
	**Sponsor PDPs**	6
	**Sponsors pharma**	6
	**Study coordinators**	12
	**Total**	40
**Experience in clinical research**		
	**<5 years**	6
	**5-10 years**	12
	**≥10 years**	22
	**Total**	40
**Mode of interviews**		
	**Face-to-face**	21
	**Online**	19
	**Total**	40

**Respectively 8 and 8 from the sponsors and the University Hospital Basel (USB).*

3.1. Status of DCTs in the Visited Research Sites

EDC, telephone calls and home visits were the main digital and decentralised solutions used in the visited sites. All the ongoing studies were using electronic data collection tools. In turn, none of the interviewees in sub-Saharan African sites had so far used wearable sensor devices for remote monitoring of clinical trials participants. At community level, home visits were conducted in the sites with the support of community health workers, as evidenced by the following statement: *"Basically, we are working with fieldworkers who perform home visits when we do not want to invite the participant to the site"* (male, investigator, Burkina Faso).

Interviewees' perceptions on the potential of DCTs were mixed, and hence, should be interpreted in context. Although most of the investigators and sponsors interviewed perceived DCTs as an asset for clinical research in sub-Saharan Africa, they stressed the need to address the challenges for their adoption and implementation.

3.2. Opportunities for DCTs

The opportunities for DCTs implementation were grouped into four main themes.

For peer review only

For peer review only - http://bmjopen.bmj.com/site/about/guidelines.xhtml

3.2.1. Site Empowerment and Sustained Site-Sponsor Collaboration

DCTs offered the possibility to pursue ongoing clinical trials especially during the COVID-19 pandemic. Despite travel restrictions during the pandemic (both international and domestic travels), the visited sites did not pause their ongoing trials for long periods (less than 6 months). The monitoring visits performed remotely by the CROs during the pandemic were perceived as efficient as the conventional approach. Owing to their clinical research experience with the sponsors, the sites experienced a shift towards more delegated tasks allowing them to take more responsibilities within the trials. This experience was perceived successful, as indicated by a principal investigator: *"COVID-19 has ensured that this development [DCTs] is evolving and we should explore it and move forward in this area. We have already started, and it is very successful. For example, in this study of [compound] conducted with [pharmaceutical company], that we completed – it was all virtual. [...] we do not have any difference. The sponsor knows our institution already – so, for experienced centres, it remains a possibility"* (male, principal investigator, Mali).

3.2.2. Improved Time and Cost-Efficiency

CROs, investigators, and sponsors perceived DCTs as an optimal approach for cost optimisation and timesaving when conducting clinical trials. The increased adoption of virtual meetings and training activities during the COVID-19 pandemic reinforced a need of change in current trial operations. A senior CRO manager emphasised the time, energy and cost savings through travel reduction: *"Some of the trainings could be done virtually, which probably saves some travel, so there is definitely a cost-efficiency that can be real"* (male, CRO manager, Kenya).

However, these perceptions are controversial for some sponsors and investigators arguing the potential drawbacks of exclusive virtual engagement with teams, stressing that there is no real overall "cost-saving" since more investments and time will be needed to finalise the trial due to quality issues. Furthermore, there is a downturn of experience sharing opportunities, as mentioned by a senior sponsor representative: *"You can conduct trainings and meetings virtually, but you also loose the experience sharing. It does not seem that it would be less expensive because you do not have to pay for the flights to these people, but in the end, it will not decrease the costs of the study because it will delay the reporting"* (male, sponsor staff, Switzerland).

3.2.3. Trial Burden

Investigators and sponsors supported that DCTs could decrease the burden of trials on participants in terms of travel and time, particularly for participants living in difficult-to-reach areas. This could improve the geographic diversity in participants' recruitment. *"- It can be a*

For peer review only - http://bmjopen.bmj.com/site/about/guidelines.xhtml

way to reduce the burden on patients in trials. They don't have to come to the site, five times" (female, sponsor staff, Switzerland). Similarly, for the sites, EDC was also perceived to alleviate the burden of data entry after the data collection.

3.2.4. Trial Quality, Documentation Improvement and Efficiency

DCTs promoted hybrid or centralised monitoring as well as remote and real-time access to the data allowing for timely corrective measures. This perception was also shared by CRO managers to speed-up trial monitoring. Digitalised documentation, electronic case report forms (eCRF) and trial files (investigator site file and trial master files) facilitated the continuity of clinical trials during the COVID-19 pandemic. Investigators reported that three ongoing studies successfully changed their master files from paper-based to electronic in the sites. *"Being able to do an electronic case report form and electronic trial master file improves efficiency. It might be a bit more expensive than a paper set-up, but it helps because you get your data in-house faster. I think there's a positive link between technology and clinical trials, and it can only get better"* (male, sponsor representative, Burkina Faso).

However, some interviewees, particularly sponsor's quality responsibles, stressed that the trend towards the digitalisation of all the processes of clinical trials with the use of various electronic platforms and systems could also become burdensome and a threat to trial quality. Hence, integrated and/or interoperable systems will be needed to avoid additional workload and errors due to loss of data or operational inaccuracies: *"If you have different suppliers managing that trial [DCTs], then those different suppliers will most likely come up with their own systems [data management, randomisation, lab supplies, CTA management]. There will be several systems in a given trial […] that are not associated with each other at all"* (male, sponsor representative, South Africa).

All interviewed sponsors perceived DCTs as an opportunity to open the discussion on innovation in clinical research, especially after the lessons learned from trials conducted during the COVID-19 pandemic. The interviewees stressed the need of innovative tools to ensure trial quality and considered DCTs as a promising option. *"There will be lessons to be learned, especially about the way we conduct trials, and we may have to think about new ways of ensuring quality"* (female, sponsor representative, Switzerland).

3.3. Main Challenges to the Digitalisation of Trials in sub-Saharan Africa

Challenges for digital transformation in clinical trials are multifactorial. The main identified challenges were grouped under four categories and included (i) the usability and practicability of the technology; (ii) paradigm shift and trial data quality; (iii) ethics and regulations; and (iv) site contextual factors (sociocultural, site specific challenges and participant literacy) (*Figure 1*).

For peer review only

For peer review only - http://bmjopen.bmj.com/site/about/guidelines.xhtml

3.3.1. Usability and Practicability of the Technology

The ability of the investigators and participants to master novel technologies within their daily work was raised as a fundamental challenge. On another note, the practical use of the technology and the lack of stable internet connectivity in some rural settings of sub-Saharan Africa were also mentioned as key challenges. The maintenance of the digital tools could be complex too, as was mentioned by some investigators: *"When you collect the data electronically – when something goes wrong, how will you take care of it? There are few things that need to happen before we can do it"* (male, study coordinator, Burkina Faso).

Moreover, the initial set-up costs, the robustness, easiness of use and the acceptability (especially for wearable devices) were also mentioned as essential barriers to be address.

3.3.2. Paradigm Shift and Trial Data Quality

Shift from conventional clinical trials to DCTs

Fully DCTs will require a complete reorganisation and change of site processes, which means a substantial shift in clinical trial paradigm. *"It's true that we may be moving towards that [DCTs], but in reality there will be a lot of efforts to bring people up to speed in order to achieve that. I dare to believe that COVID-19 or other similar events will not stop the usual process that we know"* (male, principal investigator, Mali).

On one hand, interviewed investigators stressed that the change from conventional clinical trial operations to DCTs could jeopardize the quality of clinical trials at the initiation phase and pose a threat to data validity. They suggested that changes should be limited and they would prefer the conventional or hybrid DCTs. Hence, a careful and timely planning for a consistent digital transformation is necessary. *"After COVID-19, we should go back to the old system, we should not digitalise everything"* (male, study coordinator, Mali).

On the other hand, investigators stressed the ability of sites to adjust to new technologies if there is sufficient information about these tools. *"We have been able to collect data electronically, and we have not had any problems. We can adapt in the same way as we have adapted to electronic case report forms and others"* (male, principal investigator, Burkina Faso).

Shift from a site centric to patient centric

Both sponsors and investigators also stressed the participant centric nature of the DCTs as a challenge, particularly in settings with modest literacy rates: *"I don't think it's impossible, but it's going to be difficult, because we're in a context where this type of study [full DCTs] involves the participant much more in the implementation of the trial, which assumes a certain level of literacy. It's going to be quite complicated at the moment"* (male, principal investigator,

For peer review only - http://bmjopen.bmj.com/site/about/guidelines.xhtml

Burkina Faso). However, some investigators stressed that this perception could be mitigated with adequate information and instructions about the tools intended for participants use.

3.3.3. Ethics and Regulations

The fit of DCTs within clinical trial regulations and their acceptance by ethics committees and regulatory authorities were identified as obstacles by investigators. The discomfort resulting from the use of some technologies and the challenge for ethical clearance in the countries were the main arguments suggested. This is reflected in the following statement regarding wearable devices: *"I think that ethically this will cause problems with the ethics committees because it's like an additional hygiene that we impose to the patients, if you have to wear a "heart monitor" for 24 hours, plus two or three other things, it won't be easy"* (male, principal investigator, Mali).

However, some investigators suggested mitigation strategies to address this challenge. These strategies included adequate communication, education of participants and investigators, and regulatory authority bodies regarding the risk-benefit assessment of DCTs.

3.3.4. Contextual Factors

Site-specific research environment

Site experience in clinical research is key for implementation of DCTs. The main research focus in the visited African institutions was malaria, which could be treated in ambulatory care when uncomplicated without a long-term follow-up. This trend of therapeutic area specificities in our study was foreseen and may be viewed as a limitation for some aspects of DCTs implementation. In this context, some aspects of DCTs (remote participants follow-up) were mentioned to be beneficial only for long-term follow-up data collection. *"Most of the measurements are done in the clinic. I don't think we can take that approach [full DCTs] with a malaria trial during the acute phase. In post-treatment, possibly, there might be a place for gathering adverse event information. Honestly, it's probably easier to have a healthcare worker or a nurse from the site visiting the community and gathering that information"* (female, sponsor representative, Switzerland).

Sociocultural aspects

Clinical trials' social acceptability and populations' adherence were perceived as barriers to the implementation of DCTs, as indicated by the notion of a respondent: *"The main challenge for us will be how to convince the patient"* (female, study coordinator, Burkina Faso).

Furthermore, interviewees also mentioned that the tools could create misunderstanding, trust disruption and dampen population participation in future clinical trials. Adequate and timely communication was suggested to address this issue. *"I think that the socio-cultural*

For peer review only - http://bmjopen.bmj.com/site/about/guidelines.xhtml

context can also be a blocking factor. If we use these technologies, this can initially break the existing trust between researchers and populations" (male, study coordinator, Mali).

Both sponsors and investigators also mentioned the human aspect and the risk of disruption of the relationship between investigators (as care providers) and participants. *"Physician-to-patient relationship is very important. We should not dehumanise trials as long as it is possible to do trials without putting investigators and volunteers at risk"* (male, principal investigator, Mali).

4. Discussion

We assessed the opportunities and challenges of DCTs in sub-Saharan Africa from the perspectives of CROs, investigators and sponsors. In light of these perspectives, we discuss potential solutions for efficient DCTs transformation. It is worth noticing that the interviewed stakeholders shared similar perspectives about DCTs in sub-Saharan Africa. Moreover, the research sites had consistent perspectives on DCTs implementation in their research context. Despite the multifactorial challenges reported by the interviewees, the benefits and potential of DCTs are important for clinical research improvement in sub-Saharan Africa. Several considerations are important for an efficient transition to DCTs in sub-Saharan Africa.

4.1. DCTs Technology Mastering and Context Adaptation

Some aspects of the digitalisation such as the use of wearable devices for decentralised participant monitoring remain challenging. Adaptability and acceptability are important considerations in the process of transformation from conventional clinical trials to DCTs. Recently, the adoption of DCTs during the COVID-19 pandemic was essential to ensure the continuity of ongoing trials on various medical indications, initiate COVID-19-related trials (25-27) and maintain sponsor-CROs interactions (28). The abilities of all the stakeholders including research teams, sponsors, regulatory authorities, ethics committees and participants, to master the DCTs components is essential for the transition. Sponsors, sites and, where applicable, the supporting CROs should perform an early and appropriate feasibility assessment, including site capacities and context prior to conduct DCTs and suggest adaptive improvements where necessary. User-friendly and multifunctional digital solutions (with possibility to measure several parameters, e.g. pulse, temperature and respiratory rate with one device) developed in sub-Saharan Africa and/or pilot testing are warranted. The multiplicity of the platforms used in DCTs for data acquisition and trial operations may become burdensome to the research teams and participants. Hence, these platforms should be carefully selected with guidance from investigators and participants who are the end users. Furthermore, the integration and interoperability of these platforms is required(29). The availability or development of local technical expertise for the technology maintenance will be

For peer review only - http://bmjopen.bmj.com/site/about/guidelines.xhtml

key for a long-term continuity of DCTs. Thus, the commitment of sponsors to support sites for initial investment and the continuous training will be essential.

The clinical research landscape in sub-Saharan Africa is still by far dominated by infectious diseases with most of them being acute illnesses except particularly for tuberculosis and HIV. As the research institutions in this region are adapting to address the epidemiological transition with the rising burden of chronic and non-communicable diseases, DCTs are expected to play an important role.

4.2. From Site Centric to Participant Centric Approaches

Patient-centric technologies should be context adapted. In fully DCTs, participants have important responsibilities for data collection, outcome reporting and/or clinical outcome assessment. These activities were previously conducted onsite by the research teams and sometimes with the support of community health workers. The shift from site-centric to participant-centric trials could be challenging and the type of data to be “outsourced” to the patient should be critically assessed within the context. Electronic patient reported outcomes are an important component of this change. A substantial training and clear information of trial participants and investigators and a well-defined validation process of the patient derived data is necessary. This shift also brings up the question about the compensation of research participants’ time and direct costs of the technology use since they are taking over some of the task of the research team (30). To mitigate acceptance issues and increase participants’ buy-in, patient groups should be involved in the selection of patient-centred technologies whenever possible to enhance the success of DCTs (31, 32). When possible, DCTs should be integrated as a part of patients’ follow-up with the support of their caregivers (health professionals) in the routine health facilities/hospitals. In turn, this will reinforce the research teams and caregivers collaboration. This collaboration with peripheral health facilities and hospitals of the research catchment area with clear tasks delegation can mitigate the reduced direct, face-to-face interactions with the research team. Lastly, it is important to ensure that the mentioned shift of responsibility to the patients will not waive or delay the medical care and the trial oversight by the principal investigator as well as the sponsor’s responsibilities. In this light, priority should be given to transparent communication, the interaction between investigators and participants, and the participants’ ability to attend and meet the study team anytime during trial participation.

4.3. DCTs for Clinical Trial Efficiency and Quality Improvement

The ultimate goal of DCTs is to improve trial quality and efficiency (processes, costs and duration). For instance, although broadly in use, EDC still bears the challenge of source data availability for verification and data reconciliation. This gap and the lack of clear guidelines for the tools in use as source data can also elicit ethics and regulatory authority’s reluctance or

For peer review only

For peer review only - http://bmjopen.bmj.com/site/about/guidelines.xhtml

hesitancy for the transition to DCTs. The challenge of missing data in DCTs due to poor quality of participant recordings or lack of interoperability should be addressed (33). The research team should ensure that the data reporting is similar and standardised for all the participants. The risk of underreporting or excessive reporting of adverse events for instance will be variable across participants. This could be an important threat to the trial data validity (30). Hence, a clear validation process and regular monitoring of the data collected are required. In the sub-Saharan Africa context, decentralised data collection with regular home visits by trial staff for participants monitoring or drug delivery combined with EDC, could be an alternative solution. Recently, hybrid DCTs approach was proven to be more efficient than fully onsite data collection (34). Moreover, the conventional recruitment strategy with direct engagement with the community is also proven to be efficient with a low rate of loss to follow-up (35); this should not be waved with DCTs. A hybrid recruitment model with media and online advertisement of trials on social media (36), combined with the on-site community engagement also warrants further consideration.

4.4. Ethical and Regulatory Considerations to DCTs

With regard to the ethical and regulatory considerations for DCTs, one interesting aspect raised by the investigators was a potential threat that could arise from the use of the multiplicity of digital tools, particularly for patient reporting outcomes or clinical outcome assessment. The extent of change to the participant daily habits and hygiene due to the use of digital technologies during a trial should be assessed and participants well informed before participation in a trial. This could potentially be a threat to participants' well-being as recently reported during an assessment of the ethical challenges and opportunities for DCTs (30). Other critical aspects related to the electronic informed consent and participants privacy were further mentioned (30). This is also relevant in our research context in sub-Saharan Africa where the informed consent is often challenging (37).

On another note, the need of sub-Saharan African ethics and regulatory authorities' guidance and adoption for the DCTs deployment is essential. The COVID-19 pandemic has fostered the deployment of DCTs and even decentralised performance of regulatory authorities functions such as inspections during the pandemic (38). Hence, DCTs' were addressed by the US Food and Drug Administration (FDA) (39) and the EMA and their implementation was encouraged. These guidelines triggered pharmaceutical sponsors' interest in DCTs, which was previously dominated by investigator-initiated trials (40). However, in sub-Saharan Africa, as the African Medicine Agency (AMA) and regional African Medicines Regulatory Harmonisation authorities were not fully operating, most of national regulatory authorities have thus far not revised their guidelines. This contributed to the gap in DCT implementation and their oversight by both ethics and regulatory authorities (41). Investigators and sponsors could play an

For peer review only - http://bmjopen.bmj.com/site/about/guidelines.xhtml

important role for regulatory and ethics bodies by providing them with updated information and training on available digital and decentralised solutions (8). Furthermore, study protocols should specifically mention the nature, risk-benefit and the rationale of the use of DCTs. This will contribute to mitigate the potential breaches of clinical trials' core principles, particularly the participants' protection (42, 43).

Several important questions remain as to whom has access, control and ownership of the trial data, and the storage place of the data. These issues were partially addressed by the current ICH E6 R2 requirements empowering the sites and investigators (14). DCTs should not waive these rights from the sites. Trustful, clear data storage and sharing policy should be adopted in countries where these are missing, in addition to the fulfilment of other ICH requirements.

4.5. Strengths and Limitations

This study aimed at filling a current knowledge gap pertaining to the opportunities and challenges of DCTs in sub-Saharan Africa. As the data were collected during the COVID-19 pandemic, the thriving of DCTs in this period allowed the participants to better appraise the DCTs in their context. The study was conducted in experienced, well-equipped clinical trial sites with a diversified research portfolio. However, the interviewed professionals and visited sites did not yet have broad experience with digital solutions used in clinical trials, which could have limited their perception of these technologies. The focus on CROs, investigators and sponsors did not allow us to cover the study participants' perspectives and experience that might be different.

5. Conclusion

The transition to DCTs in sub-Saharan Africa has evolved in the post-COVID-19 era. DCTs facilitated the continuity of clinical trials during the pandemic and enhanced the tasks shift from the investigators to patients. If appropriately implemented, DCTs could serve as important means to improve clinical trial efficiency. However, it is essential to assess the research context including the rationale, risks and benefits and adaptability before the deployment. The commitment of ethics committees, investigators, sponsors, and regulatory authorities, and the buy-in of the communities are essential for a successful DCT adoption. There is a need for substantial technological and logistic investment, training and communication. As DCTs are patient centric, solid and contextualised considerations with regard to good participant-investigator relationships and mutual trust should inform and guide the DCTs transition process.

Acknowledgements

For peer review only

For peer review only - http://bmjopen.bmj.com/site/about/guidelines.xhtml

We express our deep gratitude to all the interviewees and sites (Malaria Research and Training Centre- Parasitology in Mali, the Unité de Recherche Clinique de Nanoro in Burkina Faso, the University Hospital Basel in Switzerland) and the “African Contribution to Global Health” teams for their support. We are grateful to our scientific advisory committee members for their support.

Authors’ Contributions

EIN contributed to the study design, data collection, including interviews, data analysis and wrote the first draft. CB contributed to the design, data analysis, manuscript writing and review. HNS contributed to data analysis and manuscript review. PvE provided methodological guidance and contributed to the manuscript writing and review. ER, AS and JU contributed to the manuscript writing and review.

Ethical Clearance

The study was approved by the Comité d’Éthique pour la Recherche en Santé (CERS) in Burkina Faso (Decision N° 2020-12-281) and the Comité National d’Éthique in Mali (Decision 2020/287/CE/FMOS/FAPH). Additionally, the study was approved by the research commission of the Swiss Tropical and Public Health Institute (Swiss TPH; Allschwil, Switzerland), and an exemption was obtained from the Ethics Committee of Northwestern and Central Switzerland (EKNZ) (Req.2020-01364). All interview records were kept on a server with secured access to maintain confidentiality.

Funding

This study was funded by the Swiss National Science Foundation (SNSF; Bern, Switzerland) within the project “African Contribution to Global Health” (grant number CRSII5_183577).

Competing interests

None declared

Data availability statement

The data are available and could be shared upon reasonable request.

For peer review only

For peer review only - http://bmjopen.bmj.com/site/about/guidelines.xhtml

References

1. Manyazewal T, Woldeamanuel Y, Blumberg HM, et al. The potential use of digital health technologies in the African context: a systematic review of evidence from Ethiopia. NPJ Digit Med. 2021;4(1):125.

2. Banks MA. Core Concept: In the wake of COVID-19, decentralized clinical trials move to center stage. Proc Natl Acad Sci U S A. 2021;118(47).

3. Petrini C, Mannelli C, Riva L, et al. Decentralized clinical trials (DCTs): A few ethical considerations. Front Public Health. 2022;10:1081150.

4. Rogers A, De Paoli G, Subbarayan S, et al. A systematic review of methods used to conduct decentralised clinical trials. Br J Clin Pharmacol. 2022;88(6):2843-62.

5. Grignolo A. The Clinical Trials Transformation Initiative (CTTI). Ann Ist Super Sanita. 2011;47(1):14-8.

6. Inan OT, Tenaerts P, Prindiville SA, et al. Digitizing clinical trials. NPJ Digit Med. 2020;3(1):101.

7. Orri M, Lipset CH, Jacobs BP, et al. Web-based trial to evaluate the efficacy and safety of tolterodine ER 4 mg in participants with overactive bladder: REMOTE trial. Contemp Clin Trials. 2014;38(2):190-7.

8. Marquis-Gravel G, Roe MT, Turakhia MP, et al. Technology-Enabled Clinical Trials: Transforming Medical Evidence Generation. Circulation. 2019;140(17):1426-36.

9. Goldsack JC, Izmailova ES, Menetski JP, et al. Remote digital monitoring in clinical trials in the time of COVID-19. Nat Rev Drug Discov. 2020;19(6):378-9.

10. Blatch-Jones A, Nuttall J, Bull A, et al. Using digital tools in the recruitment and retention in randomised controlled trials: survey of UK Clinical Trial Units and a qualitative study. Trials. 2020;21(1):304.

11. Nissen J. Do digital clinical trials offer hope for patients? Popul Health Manag. 2020;23(4):273-5.

12. van Dam J, Omondi Onyango K, Midamba B, et al. Open-source mobile digital platform for clinical trial data collection in low-resource settings. BMJ Innov. 2017;3(1):26-31.

13. Peyser ND, Marcus GM, Beatty AL, et al. Digital platforms for clinical trials: The Eureka experience. Contemp Clin Trials. 2022;115:106710.

14. ICH. International council for harmonisation of technical requirements for pharmaceuticals for human use (ICH) 2022 [Available from: https://ichgcp.net/.

15. Sato T, Ishimaru H, Takata T, et al. Application of internet of medical/health things to decentralized clinical trials: development status and regulatory considerations. front Med (Lausanne). 2022;9:903188.

16. EMA. Guideline on computerised systems and electronic data in clinical trials 2023 [cited 2023 24.07.2023]. Available from: https://www.ema.europa.eu/en/search/search/type/ema_document?search_api_views_fulltext=Guideline%20on%20computerised%20systems%20and%20electronic%20data%20in%20clinical%20trials%20%202023. Accessed 24.07.2023

For peer review only - http://bmjopen.bmj.com/site/about/guidelines.xhtml

17. WHO. WHO Guidance for best practices for clinical trials 2023 [Available from: https://www.who.int/news-room/articles-detail/public-consultation-on-who-guidance-for-best-practices-for-clinical-trials. Accessed 24.07.2023

18. Djimde A, Ranque S, Thera MA. Building a center of excellence in biomedical research in an unfavorable environment: the Malaria Research and Training Center in Mali. Med Sante Trop. 2019;29(4):343-7.

19. van Zyl-Hermann D, Hammel T, Burri C, et al. Examining African contributions to global health: Reflections on knowledge circulation and innovation. Glob Public Health. 2022:1-16.

20. Phillippi J, Lauderdale J. A guide to field notes for qualitative research: context and conversation. Qual Health Res. 2018;28(3):381-8.

21. Kagone M, Ye M, Nebie E, et al. Community perception regarding childhood vaccinations and its implications for effectiveness: a qualitative study in rural Burkina Faso. Bmc Public Health. 2018;18.

22. De Pretto-Lazarova A, Fuchs C, van Eeuwijk P, et al. Defining clinical trial quality from the perspective of resource-limited settings: A qualitative study based on interviews with investigators, sponsors, and monitors conducting clinical trials in sub-Saharan Africa. PLoS Negl Trop Dis. 2022;16(1):e0010121.

23. Vischer N, Pfeiffer C, Kealy J, et al. Increasing protocol suitability for clinical trials in sub-Saharan Africa: a mixed methods study. Glob Health Res Policy. 2017;2:11.

24. Vischer N, Pfeiffer C, Limacher M, et al. "You can save time if..."-A qualitative study on internal factors slowing down clinical trials in Sub-Saharan Africa. PLoS One. 2017;12(3):e0173796.

25. GlobalData Healthcare. The dynamics of clinical research in Africa: 2016-2021 2022 [Available from: https://www.clinicaltrialsarena.com/comment/clinical-research-africa-2016-21/. Accessed 24.07.2023

26. Afroz MA, Schwarber G, Bhuiyan MAN. Risk-based centralized data monitoring of clinical trials at the time of COVID-19 pandemic. Contemp Clin Trials. 2021;104:106368.

27. Yamada O, Chiu SW, Takata M, et al. Clinical trial monitoring effectiveness: Remote risk-based monitoring versus on-site monitoring with 100% source data verification. Clin Trials. 2021;18(2):158-67.

28. Xue JZ, Smietana K, Poda P, et al. Clinical trial recovery from COVID-19 disruption. Nat Rev Drug Discov. 2020;19(10):662-3.

29. Clinical research in resource-limited settings. A consensus by a CIOMS Working Group., (2021).

30. Vayena E, Blasimme A, Sugarman J. Decentralised clinical trials: ethical opportunities and challenges. Lancet Digit Health. 2023;5(6):e390-e4.

31. Dockendorf MF, Hansen BJ, Bateman KP, et al. Digitally Enabled, Patient-Centric Clinical Trials: Shifting the Drug Development Paradigm. Clin Transl Sci. 2021;14(2):445-59.

32. Mowlem FD, Tenaerts P, Gwaltney C, et al. Regulatory Acceptance of Patient-Reported Outcome (PRO) Data from Bring-Your-Own-Device (BYOD) Solutions to Support Medical

For peer review only - http://bmjopen.bmj.com/site/about/guidelines.xhtml

Product Labeling Claims Let's Share the Success Stories to Move the Industry Forward. Therapeutic Innovation & Regulatory Science. 2022;56(4):531-5.

33. Di J, Demanuele C, Kettermann A, et al. Considerations to address missing data when deriving clinical trial endpoints from digital health technologies. Contemp Clin Trials. 2022;113:106661.

34. Curtis A, Qu Y. Impact of Using A Mixed Data Collection Modality on Statistical Inferences in Decentralized Clinical Trials. Ther Innov Regul Sci. 2022;56(5):744-52.

35. Idoko OT, Owolabi OA, Odutola AA, et al. Lessons in participant retention in the course of a randomized controlled clinical trial. BMC Res Notes. 2014;7:706.

36. Gunturu KS, Dizon DS, Johnson J, et al. Clinical trials in the era of digital engagement: a SWOG call to action. JCO Clin Cancer Inform. 2020;4:254-8.

37. Busisiwe N, Seeley J, Strode A, et al. Beyond translations, perspectives for researchers to consider to enhance comprehension during consent processes for health research in sub-saharan Africa: a scoping review. BMC Med Ethics. 2023;24(1):43.

38. Demasi M. FDA oversight of clinical trials is "grossly inadequate," say experts. BMJ (Clinical research ed). 2022;379:o2628.

39. FDA. Decentralized clinical trials for drugs, biological products, and devices - Guidance for industry, investigators, and other stakeholders: Silver Spring; 2020 [Available from: https://www.fda.gov/regulatory-information/search-fda-guidance-documents/decentralized-clinical-trials-drugs-biological-products-and-devices. Accessed 24.07.2023

40. Deplanque D, Cviklinski S, Bardou M, et al. Health crisis: What opportunities for clinical drug research? Therapie. 2022;77(1):59-67.

41. Solarin O, Mohammed SI, Ndlovu N, et al. Partnerships and Collaborations: The Right Alliances for Clinical Trials in Africa. JCO Glob Oncol. 2020;6:954-8.

42. van Rijssel TI, de Jong AJ, Santa-Ana-Tellez Y, et al. Ethics review of decentralized clinical trials (DCTs): Results of a mock ethics review. Drug Discov Today. 2022;27(10):103326.

43. de Jong AJ, van Rijssel TI, Zuidgeest MGP, et al. Opportunities and Challenges for Decentralized Clinical Trials: European Regulators' Perspective. Clin Pharmacol Ther. 2022;112(2):344-52.

Figure Legend:

Figure 2 Challenges for decentralised clinical trials in sub Saharan Africa

For peer review only

For peer review only - http://bmjopen.bmj.com/site/about/guidelines.xhtml

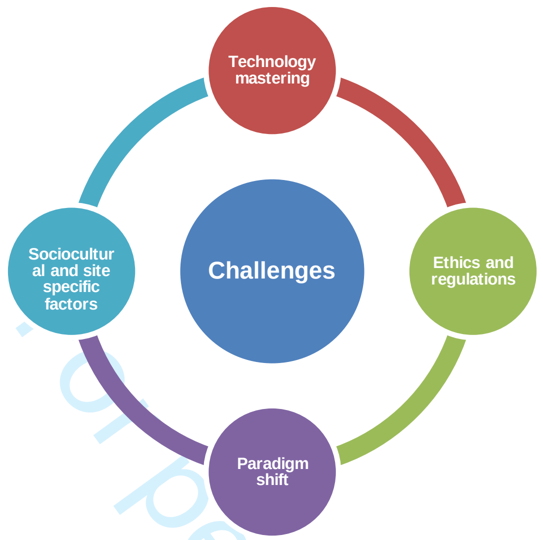

Figure 1: Challenges for decentralized clinical trials in sub-Saharan Africa

For peer review only

Standards for Reporting Qualitative Research (SRQR)*

http://www.equator-network.org/reporting-guidelines/srqr/

Title and abstract

	Page/line no(s).
**Title** - Concise description of the nature and topic of the study Identifying the study as qualitative or indicating the approach (e.g., ethnography, grounded theory) or data collection methods (e.g., interview, focus group) is recommended	Page 1 Lines 1-2
**Abstract** - Summary of key elements of the study using the abstract format of the intended publication; typically includes background, purpose, methods, results, and conclusions	Page 2 Lines 16 - 41

Introduction

**Problem formulation** - Description and significance of the problem/phenomenon studied; review of relevant theory and empirical work; problem statement	Pages 4 - 5 Lines 57 – 96
**Purpose or research question** - Purpose of the study and specific objectives or questions	Page 5 Lines 94 - 96

Methods

**Qualitative approach and research paradigm** - Qualitative approach (e.g., ethnography, grounded theory, case study, phenomenology, narrative research) and guiding theory if appropriate; identifying the research paradigm (e.g., postpositivist, constructivist/ interpretivist) is also recommended; rationale**	Page 5- 6 Lines 114 - 138
**Researcher characteristics and reflexivity** - Researchers' characteristics that may influence the research, including personal attributes, qualifications/experience, relationship with participants, assumptions, and/or presuppositions; potential or actual interaction between researchers' characteristics and the research questions, approach, methods, results, and/or transferability	Page 6 - 7 Lines 164 - 183
**Context** - Setting/site and salient contextual factors; rationale**	Page 5 Lines 100 – 111
**Sampling strategy** - How and why research participants, documents, or events were selected; criteria for deciding when no further sampling was necessary (e.g., sampling saturation); rationale**	Page 5 Lines 119 - 126
**Ethical issues pertaining to human subjects** - Documentation of approval by an appropriate ethics review board and participant consent, or explanation for lack thereof; other confidentiality and data security issues	Page 17 Lines 532 - 538
**Data collection methods** - Types of data collected; details of data collection procedures including (as appropriate) start and stop dates of data collection and analysis, iterative process, triangulation of sources/methods, and modification of procedures in response to evolving study findings; rationale**	Pages 5 - 6 Lines 128 - 162

For peer review only

For peer review only - http://bmjopen.bmj.com/site/about/guidelines.xhtml

**Data collection instruments and technologies** - Description of instruments (e.g., interview guides, questionnaires) and devices (e.g., audio recorders) used for data collection; if/how the instrument(s) changed over the course of the study	Pages 5 - 6<br>Lines 128 - 162
**Units of study** - Number and relevant characteristics of participants, documents, or events included in the study; level of participation (could be reported in results)	Page 7<br>Lines 191 - 194
**Data processing** - Methods for processing data prior to and during analysis, including transcription, data entry, data management and security, verification of data integrity, data coding, and anonymization/de-identification of excerpts	Page 6<br>Lines 148 - 162
**Data analysis** - Process by which inferences, themes, etc., were identified and developed, including the researchers involved in data analysis; usually references a specific paradigm or approach; rationale**	Page 6<br>Lines 148 – 162
**Techniques to enhance trustworthiness** - Techniques to enhance trustworthiness and credibility of data analysis (e.g., member checking, audit trail, triangulation); rationale**	Page 6<br>Lines 157 – 161

Results/findings

**Synthesis and interpretation** - Main findings (e.g., interpretations, inferences, and themes); might include development of a theory or model, or integration with prior research or theory	Page 7 - 13<br>Lines 214 – 382
**Links to empirical data** - Evidence (e.g., quotes, field notes, text excerpts, photographs) to substantiate analytic findings	Page 8 – 13<br>Lines 215-216; 233-238; 245-247; 252-256; 261-262; 272-276; 281-285; 289-294; 305-308; 314-317; 325-327; 331-334; 343-346; 358-363; 370-372; 375-377

Discussion

**Integration with prior work, implications, transferability, and contribution(s) to the field -** Short summary of main findings; explanation of how findings and conclusions connect to, support, elaborate on, or challenge conclusions of earlier scholarship; discussion of scope of application/generalizability; identification of unique contribution(s) to scholarship in a discipline or field	Page 13 - 16<br>Lines 379 – 492
**Limitations** - Trustworthiness and limitations of findings	Page3; Page 16<br>45-56; 494 - 503

Other

**Conflicts of interest** - Potential sources of influence or perceived influence on study conduct and conclusions; how these were managed	Page 17<br>Lines 545 – 546

For peer review only

**Funding** - Sources of funding and other support; role of funders in data collection, interpretation, and reporting	Page 17 Lines 534 - 536

*The authors created the SRQR by searching the literature to identify guidelines, reporting standards, and critical appraisal criteria for qualitative research; reviewing the reference lists of retrieved sources; and contacting experts to gain feedback. The SRQR aims to improve the transparency of all aspects of qualitative research by providing clear standards for reporting qualitative research.

**The rationale should briefly discuss the justification for choosing that theory, approach, method, or technique rather than other options available, the assumptions and limitations implicit in those choices, and how those choices influence study conclusions and transferability. As appropriate, the rationale for several items might be discussed together.

For peer review only

Reference:

O'Brien BC, Harris IB, Beckman TJ, Reed DA, Cook DA. **Standards for reporting qualitative research: a synthesis of recommendations.** *Academic Medicine*, Vol. 89, No. 9 / Sept 2014 DOI: 10.1097/ACM.0000000000000388

BMJ Open

Opportunities and challenges for decentralised clinical trials in sub-Saharan Africa: a qualitative study

Journal:	*BMJ Open*
Manuscript ID	bmjopen-2023-075903.R2
Article Type:	Original research
Date Submitted by the Author:	01-Sep-2023
Complete List of Authors:	Nebie, Eric; Centre de Recherche en Sante de Nouna, ; Swiss TPH, Medicines Implementation Research Unit<br>Sawadogo, Hélène N.; Centre de Recherche en Santé de Nouna<br>van Eeuwijk, Peter; Swiss Tropical and Public Health Institute; University of Basel<br>Signorell, Aita ; Swiss Tropical and Public Health Institute; University of Basel<br>Reus, Elisabeth; Swiss Tropical and Public Health Institute; University of Basel<br>Utzinger, Juerg; Swiss Tropical and Public Health Institute; University of Basel<br>Burri, Christian; Swiss Tropical and Public Health Institute; University of Basel
**Primary Subject Heading**:	Public health
Secondary Subject Heading:	Qualitative research
Keywords:	Clinical Trial, QUALITATIVE RESEARCH, PUBLIC HEALTH

SCHOLARONE™
Manuscripts

I, the Submitting Author has the right to grant and does grant on behalf of all authors of the Work (as defined in the below author licence), an exclusive licence and/or a non-exclusive licence for contributions from authors who are: i) UK Crown employees; ii) where BMJ has agreed a CC-BY licence shall apply, and/or iii) in accordance with the terms applicable for US Federal Government officers or employees acting as part of their official duties; on a worldwide, perpetual, irrevocable, royalty-free basis to BMJ Publishing Group Ltd ("BMJ") its licensees and where the relevant Journal is co-owned by BMJ to the co-owners of the Journal, to publish the Work in this journal and any other BMJ products and to exploit all rights, as set out in our licence.

The Submitting Author accepts and understands that any supply made under these terms is made by BMJ to the Submitting Author unless you are acting as an employee on behalf of your employer or a postgraduate student of an affiliated institution which is paying any applicable article publishing charge ("APC") for Open Access articles. Where the Submitting Author wishes to make the Work available on an Open Access basis (and intends to pay the relevant APC), the terms of reuse of such Open Access shall be governed by a Creative Commons licence – details of these licences and which Creative Commons licence will apply to this Work are set out in our licence referred to above.

Other than as permitted in any relevant BMJ Author's Self Archiving Policies, I confirm this Work has not been accepted for publication elsewhere, is not being considered for publication elsewhere and does not duplicate material already published. I confirm all authors consent to publication of this Work and authorise the granting of this licence.

For peer review only

Opportunities and challenges for decentralised clinical trials in sub-Saharan Africa: a qualitative study

Eric I. Nebié*[1,2,3], Hélène N. Sawadogo[3], Peter van Eeuwijk[1,2,4], Aita Signorell[1,2], Elisabeth Reus[1,2], Juerg Utzinger[1,2] and Christian Burri[1,2]

[1]Swiss Tropical and Public Health Institute, Kreuzstrasse 2, CH-4123 Allschwil, Switzerland
[2]University of Basel, Petersplatz 1, CH-4003 Basel, Switzerland
[3]Centre de Recherche en Santé de Nouna, Street Namory Keita, P.O. Box 02, Nouna, Burkina Faso
[4]Institute of Social Anthropology, University of Basel, Münsterplatz 19, CH-4051 Basel, Switzerland

*Corresponding author:
Eric Ipyn Nebié
Centre de Recherche en Santé de Nouna, Street Namory Keita, P.O. Box 02, Nouna, Burkina Faso
Swiss Tropical and Public Health Institute, Kreuzstrasse 2, CH-4123 Allschwil, Switzerland
Email: neric83@yahoo.fr

For peer review only

Abstract

Introduction

Digital health has gained traction in research and development, and clinical decision support systems. The COVID-19 pandemic accelerated the adoption of decentralised clinical trials (DCTs) as a mitigation and efficiency improvement strategy. We assessed the opportunities and challenges of a digital transformation in clinical research in sub-Saharan Africa from different stakeholders' perspectives.

Methods

A qualitative study, including 40 in-depth semi-structured interviews, was conducted with investigators of three leading research institutions in sub-Saharan Africa and Switzerland, contract research organisations and sponsors managing clinical trials in sub-Saharan Africa. A thematic approach was used for the analysis.

Results

Interviewees perceived DCTs as an opportunity for trial efficiency improvement, quality improvement and reducing the burden of people participating in clinical trials. However, to gain and maintain an optimal quality of clinical trials, a transition period is necessary to tackle contextual challenges before DCTs are being implemented. The main challenges are categorised into four themes: (i) usability and practicability of the technology; (ii) paradigm shift and trial data quality; (iii) ethical and regulatory hurdles; and (iv) contextual factors (site-specific research environment and sociocultural aspects).

Conclusion

The transformation from a site to a patient centric model with an increased responsibility of participants should be context adapted. The transformation requires substantial investment, training of the various stakeholders and an efficient communication. Additionally, commitment of sponsors, investigators, ethics, and regulatory authorities and the buy-in of the communities are essential for this change.

Keywords: Clinical research; clinical trials; decentralised clinical trials; digital health; qualitative research sub-Saharan Africa

For peer review only

Strengths and limitations

- This study provides insights into opportunities and challenges of digitalisation and decentralisation of clinical trials in sub-Saharan Africa from the perspectives and experience of investigators, contract research organisations and sponsors.
- The study was conducted in well-established research institutions, characterised by diversified research portfolios pertaining to several therapeutic areas and research context and experienced investigators.
- The conduct of the study after the onset of the COVID-19 pandemic and the increased adoption of alternative digital and decentralised solutions in research was an asset.
- This study did not include opinions of study participants who might have different perceptions than clinical trial investigators, contract research organisation and sponsors.

For peer review only

1. Introduction

Digital health technologies (DHT) are prominent innovations in global health as they hold promise to improve routine health care and foster the implementation of new health interventions (1). Decentralised clinical trials (DCTs) refers to the remote conduct of trial related activities out of the research site/clinic (2). The implementation of DCTs focuses on the use of DHT (3) and include all remote engagement with trial participants even without the DHT. The concept of DCTs is participant-centred (2). DCTs are being developed as a promising solution for future clinical research (4) to address the productivity crisis in drug research and development with continuously increasing costs, duration, burden and complexity of clinical trials (5, 6). The first documented exclusive DCT (i.e., without any participant visit to the clinic), was the “REMOTE trial”, which provided the basis for future DCTs (7). Prior to the COVID-19 pandemic, electronic data capture (EDC), electronic health records and telemedicine were the main digitalised areas in clinical trials. Recently, the use of wearable devices for electronic patient reported outcome (ePRO) and electronic clinical outcome assessment (eCOA), as well as artificial intelligence (AI), were gaining interest (8). The COVID-19 pandemic forcedly shed light on the opportunities for fast-track medical product development, while ensuring continuity of trials under the pandemic’s stringent restrictions (9). DCTs have proven to be feasible and to have potential to improve participant recruitment, retention and representability in clinical trials (10, 11), data collection (12) and efficiency (13). However, in a recent systematic review covering studies conducted in Asia, Australia, Europe and North America, there was little consensual added value of digitalisation in clinical research (4).

Before the COVID-19 pandemic, DCTs were not fully covered by ethics and regulatory guidelines. Guidance on electronic records and electronic signatures were first included into the US Code of Federal Regulations Title 21, Part 11 (21 CFR Part 11). The first version and the revision 2 of the International Conference on Harmonization (ICH) Guideline E6 Good Clinical Practices (GCP) (14) mentioned the use of digital and decentralised solutions in a few instances; yet, in a rather superficial way. Hence, the adoption of DCTs was challenging. By February 2022, only two countries (Denmark and Switzerland) had digital clinical trials included in their guidelines (15). In-depth deliberation of the topic was initiated in 2010 with a European Medicines Agency (EMA) position paper covering electronic source data and data transcribed to electronic data collection tools and was further detailed in 2023 in the Guideline on computerised systems and electronic data in clinical trials (16). The upcoming revision 3 of the ICH GCP and World Health Organization (WHO) guidance for best practices for clinical trials under public consultation, provides more details and encourages the use of DCTs to improve clinical trials efficiency (14, 17).

In sub-Saharan Africa, the transition to DCTs is at an early stage. With the outset and rapid spread of SARS-CoV-2, DCTs were expeditiously initiated in some research institutions

For peer review only - http://bmjopen.bmj.com/site/about/guidelines.xhtml

in sub-Saharan Africa to allow continuity of ongoing clinical trials despite the unfolding pandemic. The current study aimed to address the opportunities and challenges for the transition to DCTs, placing particular emphasis on sub-Saharan Africa.

2. Methods

2.1. Study Settings

Data were collected in two leading clinical research institutions in sub-Saharan Africa; the Malaria Research and Training Centre (MRTC) in Mali and the Clinical Research Unit of Nanoro (URCN) in Burkina Faso. Additionally, data were obtained from four departments of the University Hospital Basel (USB) in Switzerland that are conducting clinical trials. The USB is also involved in clinical trials conducted in various settings including sub-Saharan Africa.

The two African research institutions have a long-standing clinical research experience with various sponsors from academia, product development partnerships (PDPs) and the pharmaceutical industry. These centres have strong capacities in terms of infrastructures, equipment and human resources and comply with international standards (18). The two research institutions' catchment areas include both rural and semi-urban settings; they operate in research clinics, hospitals and communities.

2.2. Study Design

We pursued a qualitative study to identify and explore the efficiency drivers of clinical development from various stakeholders' perspectives (19). Several strategies for clinical trials efficiency were identified including study design and complexity, quality approaches (presented elsewhere) and decentralization of clinical trials. The presented work focused on the opportunities and challenges for digitalisation and decentralisation in clinical research assessed through the qualitative interviews. This topic was triggered by the escalating use of DCTs, particularly during and after the COVID-19 pandemic.

2.3. Interviews

The in-depth semi-structured interviews were conducted with clinical researchers (principal investigators and study coordinators), sponsors (project managers, project leaders, clinical development staff, quality and regulatory affairs responsible, and medical officers) and contract research organisations (CROs) managers who have been involved in at least one clinical trial in the past 3 years in sub-Saharan Africa. Three different interview guidelines were used for the investigators, sponsors and CROs. The main themes covered during the interviews were the interviewees' experience with DCTs before and during the COVID-19

For peer review only - http://bmjopen.bmj.com/site/about/guidelines.xhtml

pandemic, their perceptions of DCTs, potential benefits for sponsors and sites and, the perceived challenges for DCTs implementation.

A scientific advisory committee, comprising representatives from each sponsor type and a sub-Saharan Africa research institution leader (one from pharmaceutical industry, for profit; one from a biotech company, for profit; one from a PDP, not for profit; and one from academia), reviewed the interview guides. Three members were working in Swiss-based companies with clinical research experience in sub-Saharan Africa and one in a research institution in Burkina Faso. The committee members provided inputs based on their expertise in clinical research. They were not interviewed and did not have any direct relation with the visited sites. The study was neither commissioned nor influenced by any type of sponsor.

2.4. Data Collection and Analysis

The data were collected from December 2020 to October 2021. Interviews were either performed face-to-face (during the 6-week stay in the sites) or virtually using a Zoom license maintained by the Swiss Tropical and Public Health Institute (Swiss TPH; Allschwil, Switzerland). The interviews were conducted by the first author either in English or in French, according to the interviewees' preference. The interviews lasted between 45 and 90 min, depending on the interviewees' experience. The interviews were recorded, transcribed, translated, coded and analysed using MAXQDA version 20 (VERBI Software 2021; Berlin, Germany). A deductive and inductive thematic analysis was pursued. Two researchers (EIN and HNS) independently performed the coding after an extended reading of the transcripts. Subcodes and codes were generated and grouped into themes. Some preconceived themes were identified from the existing literature as the deductive analysis. Data were inductively analysed and new themes identified.

2.5. Researchers' Characteristics, Reflexivity and Trustworthiness

The study was conducted by researchers of Swiss TPH and the Centre de Recherche en Santé de Nouna (CRSN) in Burkina Faso. All involved researchers have extensive experience in the conduct of clinical studies. In addition, all of them had received specific trainings in qualitative research and made previous contributions to mixed method studies about the quality and efficiency of clinical studies, and immunization mainly in resource-constrained settings (20-23). The researchers did not have working experience with the sites where the study was conducted. EIN was introduced to the teams by Swiss TPH senior staff through the site leaders. Participants were given the opportunity to ask for clarifications after reading the study information sheet. The research team was aware that a commissioned study by the sponsor could induce significant bias. Hence, it was mentioned clearly to participants that the study is conducted independently and not commissioned by any sponsor. Participants felt

For peer review only - http://bmjopen.bmj.com/site/about/guidelines.xhtml

comfortable to share their views regarding the topic. EIN and HNS are based and worked in sub-Saharan Africa and have a deep understanding of the research context. The credibility was ensured by selecting participants with different roles and responsibilities in clinical trials. As the data were subsequently collected in different research sites, it was safeguarded that the interviews were not affected by the insights gained in the different contexts and the interviews were similar and comparable. The findings were discussed within the team to ensure that the results are correctly interpreted within the context. The current findings could be transferred in research environments sharing several commonalities in sub-Saharan Africa.

2.6. Patient and Public Involvement Statement

The study participants were clinical research stakeholders (investigators, CROs and sponsors); the scientific advisory committee members were involved in the review of data collection tools based on their expertise. We did not include patients in the design, conduct, reporting and dissemination plan of the study.

3. Results

Overall, 40 in-depth semi-structured interviews were conducted. Previous experience of DCTs before and after the onset of the COVID-19 pandemic, perceived context-specific DCTs opportunities and challenges, and potential solutions were discussed during the interviews. The interviewees' characteristics are summarised in Table 1.

For peer review only - http://bmjopen.bmj.com/site/about/guidelines.xhtml

Table 1: Interviewees' characteristics

Participants' profiles		Number
**Gender**		
	**Male**	29
	**Female**	11
	**Total**	40
**Country of residence**		
	**Burkina Faso**	8
	**Germany**	2
	**Kenya**	1
	**Mali**	9
	**Senegal**	1
	**South Africa**	2
	**Switzerland**	16*
	**USA**	1
	**Total**	40
**Main role**		
	**CRO managers**	4
	**Principal investigators**	12
	**Sponsor PDPs**	6
	**Sponsors pharma**	6
	**Study coordinators**	12
	**Total**	40
**Experience in clinical research**		
	**<5 years**	6
	**5-10 years**	12
	**≥10 years**	22
	**Total**	40
**Mode of interviews**		
	**Face-to-face**	21
	**Online**	19
	**Total**	40

**Respectively 8 from the sponsors and 8 from the University Hospital Basel (USB).*

3.1. Status of DCTs in the Visited Research Sites

EDC, telephone calls and home visits were the main digital and decentralised solutions used in the visited sites. All the ongoing studies were using electronic data collection tools. In turn, none of the interviewees in sub-Saharan African sites had so far used wearable sensor devices for remote monitoring of clinical trials participants. At community level, home visits were carried out with the support of community health workers, as evidenced by the following statement: *"Basically, we are working with fieldworkers who perform home visits when we do not want to invite the participant to the site"* (male, investigator, Burkina Faso).

Interviewees' perceptions on the potential of DCTs were mixed, and hence, should be interpreted in context. Although most of the investigators and sponsors interviewed perceived DCTs as an asset for clinical research in sub-Saharan Africa, they stressed the need to address the challenges for their adoption and implementation.

3.2. Opportunities for DCTs

The opportunities for DCTs implementation were grouped into four main themes.

For peer review only

For peer review only - http://bmjopen.bmj.com/site/about/guidelines.xhtml

3.2.1. Site Empowerment and Sustained Site-Sponsor Collaboration

DCTs offered the possibility to pursue ongoing clinical trials especially during the COVID-19 pandemic. Despite travel restrictions during the pandemic (both international and domestic travels), the visited sites did not pause their ongoing trials for long periods (less than 6 months). The monitoring visits performed remotely by the CROs during the pandemic were perceived as efficient as the conventional approach. Owing to their clinical research experience with the sponsors, the sites experienced a shift towards more delegated tasks allowing them to take more responsibilities within trials. This experience was perceived successful, as indicated by a principal investigator: *"COVID-19 has ensured that this development [DCTs] is evolving and we should explore it and move forward in this area. We have already started, and it is very successful. For example, in this study of [compound] conducted with [pharmaceutical company], that we completed – it was all virtual. [...] we do not have any difference. The sponsor knows our institution already – so, for experienced centres, it remains a possibility"* (male, principal investigator, Mali).

3.2.2. Improved Time and Cost-Efficiency

CROs, investigators, and sponsors perceived DCTs as an optimal approach for cost optimisation and timesaving when conducting clinical trials. The increased adoption of virtual meetings and training activities during the COVID-19 pandemic reinforced the need of change in current trial operations. A senior CRO manager emphasised the time, energy and cost savings through travel reduction: *"Some of the trainings could be done virtually, which probably saves some travel, so there is definitely a cost-efficiency that can be real"* (male, CRO manager, Kenya).

However, these perceptions are controversial for some sponsors and investigators arguing the potential drawbacks of exclusive virtual engagement with teams, stressing that there is no real overall "cost-saving" since more investments and time will be needed to finalise the trial due to quality issues. Furthermore, there is a downturn of experience sharing opportunities, as mentioned by a senior sponsor representative: *"You can conduct trainings and meetings virtually, but you will lose the experience sharing. It does not seem to be less expensive because you do not have to pay for the flights, but in the end, it will not reduce the costs of the study because it will delay the reporting"* (male, sponsor staff, Switzerland).

3.2.3. Trial Burden

Investigators and sponsors supported that DCTs could decrease the burden of trials on participants in terms of travel and time, particularly for participants living in difficult-to-reach areas. This could improve the geographic diversity in participants' recruitment. *"- It can be a way to reduce the burden of trials on patients. They don't have to come to the site, five times..."*

For peer review only - http://bmjopen.bmj.com/site/about/guidelines.xhtml

(female, sponsor staff, Switzerland). Similarly, for the sites, EDC was perceived to alleviate the burden of data entry after the data collection.

3.2.4. Trial Quality, Documentation Improvement and Efficiency

DCTs promoted hybrid or centralised monitoring as well as remote and real-time access to the data allowing for timely corrective measures. This perception was also shared by CRO managers to speed-up trial monitoring. Digitalised documentation, electronic case report forms (eCRF) and trial files (investigator site file and trial master file) facilitated the continuity of clinical trials during the COVID-19 pandemic. Investigators reported that three ongoing studies successfully changed their master files from paper-based to electronic in the sites. *"Being able to use an electronic case report form and electronic trial master file improves efficiency. It might be a bit more expensive than a paper set-up, but it helps because you get your data in-house faster. I think there's a positive link between technology and clinical trials, and it can only get better"* (male, sponsor representative, Burkina Faso).

However, some interviewees, particularly sponsor's quality responsible, stressed that the trend towards the digitalisation of all the processes of clinical trials with the use of various electronic platforms and systems could also become burdensome and a threat to trial quality. Hence, integrated and/or interoperable systems will be needed to avoid additional workload and errors due to loss of data or operational inaccuracies: *"If you have different suppliers managing that trial [DCTs], then those different suppliers will most likely come up with their own systems [data management, randomisation, lab supplies, CTA management]. There will be several systems in a given trial […] that are not connected at all"* (male, sponsor representative, South Africa).

All interviewed sponsors perceived DCTs as an opportunity to open the discussion on innovation in clinical research, especially after the lessons learned from trials conducted during the COVID-19 pandemic. The interviewees stressed the need of innovative tools to ensure trial quality and considered DCTs as a promising option. *"There will be lessons to be learnt, particularly about the way we conduct trials, and we may have to think about new ways of ensuring quality"* (female, sponsor representative, Switzerland).

3.3. Main Challenges to the Digitalisation of Trials in sub-Saharan Africa

Challenges for digital transformation in clinical trials are multifactorial. The main identified challenges were grouped under four categories and included (i) the usability and practicability of the technology; (ii) paradigm shift and trial data quality; (iii) ethics and regulations; and (iv) site contextual factors (sociocultural, site specific challenges and participant literacy) (*Figure 1*).

For peer review only - http://bmjopen.bmj.com/site/about/guidelines.xhtml

3.3.1. Usability and Practicability of the Technology

The ability of the investigators and participants to master novel technologies within their daily work was raised as a fundamental challenge. Furthermore, the practical use of the technology and the lack of stable internet connectivity in some rural settings of sub-Saharan Africa were also mentioned as key challenges. The maintenance of the digital tools could be complex too, as was mentioned by some investigators: *"When you collect the data electronically – when something goes wrong, how will you take care of it? There are few things that need to happen before we can do it"* (male, study coordinator, Burkina Faso).

Moreover, the initial set-up costs, the robustness, easiness of use and the acceptability (especially for wearable devices) were also mentioned as essential barriers to be address.

3.3.2. Paradigm Shift and Trial Data Quality

Shift from conventional clinical trials to DCTs

Fully DCTs will require a complete reorganisation and change of site processes, which means a substantial shift in clinical trial paradigm. *"It's true that we may be moving towards that [DCTs], but in reality there will be a lot of efforts to bring people up to speed in order to achieve that. I dare to believe that COVID-19 or other similar events will not stop the usual process that we know"* (male, principal investigator, Mali).

On the one hand, interviewed investigators stressed that the change from conventional clinical trial operations to DCTs could jeopardize the quality of clinical trials at the initiation phase and pose a threat to data validity. They suggested that changes should be limited and they would prefer the conventional or hybrid DCTs. Hence, a careful and timely planning for a consistent digital transformation is necessary. *"After COVID-19, we should go back to the old system, we should not digitalise everything"* (male, study coordinator, Mali).

On the other hand, investigators stressed the ability of sites to adjust to new technologies if there is sufficient information about these tools. *"We have been able to collect data electronically, and we have not had any problems. We can adapt in the same way as we have adapted to electronic case report forms and others"* (male, principal investigator, Burkina Faso).

Shift from a site centric to patient centric

Sponsors and investigators also highlighted the participant-centric nature of DCTs as a challenge, particularly in settings with modest literacy rates. *"I don't think it's impossible, but it's going to be difficult, because we're in a context where this type of study [full DCTs] involves the participant much more in the implementation of the trial, which assumes a certain level of literacy. It's going to be quite complicated at the moment"* (male, principal investigator, Burkina Faso). However, some investigators stressed that this perception could be mitigated with adequate information and instructions about the tools intended for participants use.

For peer review only - http://bmjopen.bmj.com/site/about/guidelines.xhtml

3.3.3. Ethics and Regulations

The fit of DCTs within clinical trial regulations and their acceptance by ethics committees and regulatory authorities were identified as obstacles by investigators. The discomfort resulting from the use of some technologies and the challenge for ethical clearance in the countries were the main arguments. This is reflected in the following statement regarding wearable devices: *"I think that ethically this will cause problems with the ethics committees because it's like an additional hygiene that we impose to the patients, if you have to wear a "heart monitor" for 24 hours, plus two or three other things, it won't be easy"* (male, principal investigator, Mali).

However, some investigators suggested mitigation strategies to address this challenge. These strategies included adequate communication, education of participants and investigators, and regulatory authority bodies regarding the risk-benefit assessment of DCTs.

3.3.4. Contextual Factors

Site-specific research environment

Site experience in clinical research is key for implementation of DCTs. The main research focus in the visited African institutions was malaria, which could be treated in ambulatory care when uncomplicated without a long-term follow-up. This trend of therapeutic area specificities in our study was anticipated and may be viewed as a limitation for some aspects of DCTs implementation. In this context, some aspects of DCTs (remote participants follow-up) were mentioned to be beneficial only for long-term follow-up data collection. *"Most of the measurements are done in the clinic. I don't think we can take that approach [full DCTs] with a malaria trial during the acute phase. In post-treatment, possibly, there might be a place for gathering adverse event information. Honestly, it's probably easier to have a healthcare worker or a nurse from the site visiting the community and gathering that information"* (female, sponsor representative, Switzerland).

Sociocultural aspects

Clinical trials' social acceptability and populations' adherence were perceived as barriers to the implementation of DCTs, as indicated by the notion of a respondent: *"The main challenge for us will be how to convince the patient"* (female, study coordinator, Burkina Faso).

Furthermore, interviewees also mentioned that the tools could create misunderstanding, trust disruption and dampen population participation in future clinical trials. Adequate and timely communication was suggested to address this issue. *"I think that the socio-cultural context can also be a blocking factor. If we use these technologies, this can initially break the existing trust between researchers and populations"* (male, study coordinator, Mali).

For peer review only - http://bmjopen.bmj.com/site/about/guidelines.xhtml

For peer review only

Both sponsors and investigators also mentioned the human aspect and the risk of disruption of the relationship between investigators (as care providers) and participants. *"Physician-to-patient relationship is very important. We should not dehumanise trials as long as it is possible to do trials without putting investigators and volunteers at risk"* (male, principal investigator, Mali).

4. Discussion

We assessed the opportunities and challenges of DCTs in sub-Saharan Africa from the perspectives of CROs, investigators and sponsors. In light of these perspectives, we discuss potential solutions for efficient DCTs transformation. It is worth noticing that the interviewed stakeholders shared similar perspectives about DCTs in sub-Saharan Africa. Moreover, the research sites had consistent perspectives on DCTs implementation in their research context. Despite the multifactorial challenges reported by the interviewees, the benefits and potential of DCTs are important for clinical research improvement in sub-Saharan Africa. Several considerations are important for an efficient transition to DCTs in sub-Saharan Africa.

4.1. DCTs Technology Mastering and Context Adaptation

Some aspects of the digitalisation such as the use of wearable devices for decentralised participant monitoring remain challenging. Adaptability and acceptability are important considerations in the process of transformation from conventional clinical trials to DCTs. Recently, the adoption of DCTs during the COVID-19 pandemic was essential to ensure the continuity of ongoing trials in various medical indications, initiate COVID-19-related trials (24-26) and maintain sponsor-CROs interactions (27). The abilities of all the stakeholders including research teams, sponsors, regulatory authorities, ethics committees and participants, to master DCTs components is essential for the transition. Sponsors, sites and, where applicable, the supporting CROs should perform an early and appropriate feasibility assessment, including site capacities and context prior to conduct DCTs and suggest adaptive improvements where necessary. User-friendly and multifunctional digital solutions (with possibility to measure several parameters, e.g. pulse, temperature and respiratory rate with one device) developed in sub-Saharan Africa and/or pilot testing are warranted. The multiplicity of the platforms used in DCTs for data acquisition and trial operations may become burdensome to the research teams and participants. Hence, these platforms should be carefully selected with guidance from investigators and participants who are the end users. Furthermore, the integration and interoperability of these platforms is required(28). The availability or development of local technical expertise for the technology maintenance will be key for a long-term continuity of DCTs. Thus, the commitment of sponsors to support sites for initial investment and the continuous training will be essential.

For peer review only - http://bmjopen.bmj.com/site/about/guidelines.xhtml

The clinical research landscape in sub-Saharan Africa is still by far dominated by infectious diseases with most of them being acute illnesses except for tuberculosis and HIV. As the research institutions in this region are adapting to address the epidemiological transition with the rising burden of chronic and non-communicable diseases, DCTs are expected to play an important role.

4.2. From Site Centric to Participant Centric Approaches

Patient-centric technologies should be context adapted. In fully DCTs, participants have important responsibilities for data collection, outcome reporting and/or clinical outcome assessment. These activities were previously conducted onsite by the research teams and sometimes with the support of community health workers. The shift from site-centric to participant-centric trials could be challenging and the type of data to be "outsourced" to the patient should be critically assessed within the context. Electronic patient reported outcomes are an important component of this change. A substantial training and clear information of trial participants and investigators and a well-defined validation process of the patient derived data is necessary. This shift also brings up the question about the compensation of research participants' time and direct costs of the technology use since they are taking over some of the tasks of the research team (29). To mitigate acceptance issues and increase participants' buy-in, patient groups should be involved in the selection of patient-centred technologies whenever possible to enhance the success of DCTs (30, 31). When possible, DCTs should be integrated as a part of patients' follow-up with the support of their caregivers (health professionals) in the routine health facilities/hospitals. In turn, this will reinforce the research teams and caregivers collaboration. This collaboration with peripheral health facilities and hospitals of the research catchment area with clear tasks delegation can mitigate the reduced direct, face-to-face interactions with the research team. Lastly, it is important to ensure that the mentioned shift of responsibility to the patients will not waive or delay the medical care and the trial oversight by the principal investigator as well as the sponsor's responsibilities. In this light, priority should be given to transparent communication, the interaction between investigators and participants, and the participants' ability to attend and meet the study team anytime during trial participation.

4.3. DCTs for Clinical Trial Efficiency and Quality Improvement

The ultimate goal of DCTs is to improve trial quality and efficiency (processes, costs and duration). For instance, although broadly in use, EDC still bears the challenge of source data availability for verification and data reconciliation. This gap and the lack of clear guidelines for the tools in use as source data can also elicit ethics and regulatory authority's reluctance or hesitancy for the transition to DCTs. The challenge of missing data in DCTs due to poor quality of participant recordings or lack of interoperability should be addressed (32). The research

For peer review only - http://bmjopen.bmj.com/site/about/guidelines.xhtml

team should ensure that the data reporting is similar and standardised for all the participants. The risk of underreporting or excessive reporting of adverse events for instance will be variable across participants. This could be an important threat to the trial data validity (29). Hence, a clear validation process and regular monitoring of the data collected are required. In the sub-Saharan Africa context, decentralised data collection with regular home visits by trial staff for participants monitoring or drug delivery combined with EDC, could be an alternative solution. Recently, hybrid DCTs approach was proven to be more efficient than fully onsite data collection (33). Moreover, the conventional recruitment strategy with direct engagement with the community is also proven to be efficient with a low rate of loss to follow-up (34); this should not be waved with DCTs. A hybrid recruitment model with media and online advertisement of trials on social media (35), combined with the on-site community engagement also warrants further consideration.

4.4. Ethical and Regulatory Considerations to DCTs

With regard to the ethical and regulatory considerations for DCTs, one interesting aspect raised by the investigators was a potential threat that could arise from the use of the multiplicity of digital tools, particularly for patient reported outcomes or clinical outcome assessment. The extent of change to the participant daily habits and hygiene due to the use of digital technologies during a trial should be assessed and participants well informed before participation in a trial. This could potentially be a threat to participants' well-being as recently reported during an assessment of the ethical challenges and opportunities for DCTs (29). Other critical aspects related to the electronic informed consent and participants' privacy were further mentioned (29). This is also relevant in our research context in sub-Saharan Africa where the informed consent is often challenging (36).

The need of sub-Saharan African ethics and regulatory authorities' guidance and adoption for the DCTs deployment is essential. The COVID-19 pandemic has fostered the deployment of DCTs and even decentralised performance of regulatory authorities functions such as inspections during the pandemic (37). Hence, DCTs were addressed by the US Food and Drug Administration (FDA) (38) and the EMA and their implementation was encouraged. These guidelines triggered pharmaceutical sponsors' interest in DCTs, which was previously dominated by investigator-initiated trials (39). However, in sub-Saharan Africa, as the African Medicine Agency (AMA) and regional African Medicines Regulatory Harmonisation authorities were not fully operating, most of national regulatory authorities have thus far not revised their guidelines. This contributed to the gap in DCT implementation and their oversight by both ethics and regulatory authorities (40). Investigators and sponsors could play an important role for regulatory and ethics bodies by providing them with updated information and training on available digital and decentralised solutions (8). Furthermore, study protocols should

For peer review only - http://bmjopen.bmj.com/site/about/guidelines.xhtml

specifically mention the nature, risk-benefit and the rationale of the use of DCTs. This will contribute to mitigate the potential breaches of clinical trials' core principles, particularly the participants' protection (41, 42).

Several important questions remain as to whom has access, control and ownership of the trial data, and the storage place of the data. These issues were partially addressed by the current ICH E6 R2 requirements empowering the sites and investigators (14). DCTs should not waive these rights from the sites. Trustful, clear data storage and sharing policies should be adopted in countries where these are missing, in addition to the fulfilment of other ICH requirements.

4.5. Strengths and Limitations

This study aimed at filling a current knowledge gap pertaining to the opportunities and challenges of DCTs in sub-Saharan Africa. As the data were collected during the COVID-19 pandemic, the thriving of DCTs in this period allowed the participants to better appraise DCTs in their context. The study was conducted in experienced, well-equipped clinical trial sites with a diversified research portfolio. However, the interviewed professionals and visited sites did not yet have broad experience with some digital solutions used in clinical trials, which could have limited their perception of these technologies. The focus on CROs, investigators and sponsors did not allow us to cover the study participants' perspectives and experience that might be different.

5. Conclusion

The transition to DCTs in sub-Saharan Africa has evolved in the post-COVID-19 era. DCTs facilitated the continuity of clinical trials during the pandemic and enhanced the tasks shift from the investigators to patients. If appropriately implemented, DCTs could serve as important means to improve clinical trial efficiency. However, it is essential to assess the research context including the rationale, risks and benefits and adaptability before the deployment. The commitment of ethics committees, investigators, sponsors, and regulatory authorities, and the buy-in of the communities are essential for a successful DCT adoption. There is a need for substantial technological and logistic investment, training and communication. As DCTs are patient centric, solid and contextualised considerations with regard to good participant-investigator relationships and mutual trust should inform and guide the DCTs transition process.

Acknowledgements

We express our deep gratitude to all the interviewees and sites (Malaria Research and Training Centre- Parasitology in Mali, the Unité de Recherche Clinique de Nanoro in Burkina Faso, the

For peer review only

For peer review only - http://bmjopen.bmj.com/site/about/guidelines.xhtml

University Hospital Basel in Switzerland) and the "African Contribution to Global Health" teams for their support. We are grateful to our scientific advisory committee members for their support.

Authors' Contributions

EIN contributed to the study design, data collection, including interviews, data analysis and wrote the first draft. CB contributed to the design, data analysis, manuscript writing and review. HNS contributed to data analysis and manuscript review. PvE provided methodological guidance and contributed to the manuscript writing and review. ER, AS and JU contributed to the manuscript writing and review.

Ethical Clearance

The study was approved by the Comité d'Éthique pour la Recherche en Santé (CERS) in Burkina Faso (Decision N° 2020-12-281) and the Comité National d'Éthique in Mali (Decision 2020/287/CE/FMOS/FAPH). Additionally, the study was approved by the research commission of the Swiss Tropical and Public Health Institute (Swiss TPH; Allschwil, Switzerland), and an exemption was obtained from the Ethics Committee of Northwestern and Central Switzerland (EKNZ) (Req.2020-01364). All interview records were kept on a server with secured access to maintain confidentiality.

Funding

This study was funded by the Swiss National Science Foundation (SNSF; Bern, Switzerland) within the project "African Contribution to Global Health" (grant number CRSII5_183577).

Competing interests

None declared

Data availability statement

The data are available and could be shared upon reasonable request.

For peer review only

For peer review only - http://bmjopen.bmj.com/site/about/guidelines.xhtml

References

1. Manyazewal T, Woldeamanuel Y, Blumberg HM, et al. The potential use of digital health technologies in the African context: a systematic review of evidence from Ethiopia. NPJ Digit Med. 2021;4(1):125.

2. Banks MA. Core Concept: In the wake of COVID-19, decentralized clinical trials move to center stage. Proc Natl Acad Sci U S A. 2021;118(47).

3. Petrini C, Mannelli C, Riva L, et al. Decentralized clinical trials (DCTs): A few ethical considerations. Front Public Health. 2022;10:1081150.

4. Rogers A, De Paoli G, Subbarayan S, et al. A systematic review of methods used to conduct decentralised clinical trials. Br J Clin Pharmacol. 2022;88(6):2843-62.

5. Grignolo A. The Clinical Trials Transformation Initiative (CTTI). Ann Ist Super Sanita. 2011;47(1):14-8.

6. Inan OT, Tenaerts P, Prindiville SA, et al. Digitizing clinical trials. NPJ Digit Med. 2020;3(1):101.

7. Orri M, Lipset CH, Jacobs BP, et al. Web-based trial to evaluate the efficacy and safety of tolterodine ER 4 mg in participants with overactive bladder: REMOTE trial. Contemp Clin Trials. 2014;38(2):190-7.

8. Marquis-Gravel G, Roe MT, Turakhia MP, et al. Technology-Enabled Clinical Trials: Transforming Medical Evidence Generation. Circulation. 2019;140(17):1426-36.

9. Goldsack JC, Izmailova ES, Menetski JP, et al. Remote digital monitoring in clinical trials in the time of COVID-19. Nat Rev Drug Discov. 2020;19(6):378-9.

10. Blatch-Jones A, Nuttall J, Bull A, et al. Using digital tools in the recruitment and retention in randomised controlled trials: survey of UK Clinical Trial Units and a qualitative study. Trials. 2020;21(1):304.

11. Nissen J. Do digital clinical trials offer hope for patients? Popul Health Manag. 2020;23(4):273-5.

12. van Dam J, Omondi Onyango K, Midamba B, et al. Open-source mobile digital platform for clinical trial data collection in low-resource settings. BMJ Innov. 2017;3(1):26-31.

13. Peyser ND, Marcus GM, Beatty AL, et al. Digital platforms for clinical trials: The Eureka experience. Contemp Clin Trials. 2022;115:106710.

14. ICH. International council for harmonisation of technical requirements for pharmaceuticals for human use (ICH) 2022 [Available from: https://ichgcp.net/. Accessed 24.07.2023]

15. Sato T, Ishimaru H, Takata T, et al. Application of internet of medical/health things to decentralized clinical trials: development status and regulatory considerations. front Med (Lausanne). 2022;9:903188.

16. EMA. Guideline on computerised systems and electronic data in clinical trials 2023 [cited 2023 24.07.2023]. Available from: https://www.ema.europa.eu/en/search/search/type/ema_document?search_api_views_fulltext=Guideline%20on%20computerised%20systems%20and%20electronic%20data%20in%20clinical%20trials%20%202023. Accessed 24.07.2023]

For peer review only

For peer review only - http://bmjopen.bmj.com/site/about/guidelines.xhtml

17. WHO. WHO Guidance for best practices for clinical trials 2023 [Available from: https://www.who.int/news-room/articles-detail/public-consultation-on-who-guidance-for-best-practices-for-clinical-trials. Accessed 24.07.2023].

18. Djimde A, Ranque S, Thera MA. Building a center of excellence in biomedical research in an unfavorable environment: the Malaria Research and Training Center in Mali. Med Sante Trop. 2019;29(4):343-7.

19. van Zyl-Hermann D, Hammel T, Burri C, et al. Examining African contributions to global health: Reflections on knowledge circulation and innovation. Glob Public Health. 2022:1-16.

20. Kagone M, Ye M, Nebie E, et al. Community perception regarding childhood vaccinations and its implications for effectiveness: a qualitative study in rural Burkina Faso. Bmc Public Health. 2018;18.

21. De Pretto-Lazarova A, Fuchs C, van Eeuwijk P, et al. Defining clinical trial quality from the perspective of resource-limited settings: A qualitative study based on interviews with investigators, sponsors, and monitors conducting clinical trials in sub-Saharan Africa. PLoS Negl Trop Dis. 2022;16(1):e0010121.

22. Vischer N, Pfeiffer C, Kealy J, et al. Increasing protocol suitability for clinical trials in sub-Saharan Africa: a mixed methods study. Glob Health Res Policy. 2017;2:11.

23. Vischer N, Pfeiffer C, Limacher M, et al. "You can save time if..."-A qualitative study on internal factors slowing down clinical trials in Sub-Saharan Africa. PLoS One. 2017;12(3):e0173796.

24. GlobalData Healthcare. The dynamics of clinical research in Africa: 2016-2021 2022 [Available from: https://www.clinicaltrialsarena.com/comment/clinical-research-africa-2016-21/. Accessed 23.07.2023].

25. Afroz MA, Schwarber G, Bhuiyan MAN. Risk-based centralized data monitoring of clinical trials at the time of COVID-19 pandemic. Contemp Clin Trials. 2021;104:106368.

26. Yamada O, Chiu SW, Takata M, et al. Clinical trial monitoring effectiveness: Remote risk-based monitoring versus on-site monitoring with 100% source data verification. Clin Trials. 2021;18(2):158-67.

27. Xue JZ, Smietana K, Poda P, et al. Clinical trial recovery from COVID-19 disruption. Nat Rev Drug Discov. 2020;19(10):662-3.

28. Clinical research in resource-limited settings. A consensus by a CIOMS Working Group., (2021).

29. Vayena E, Blasimme A, Sugarman J. Decentralised clinical trials: ethical opportunities and challenges. Lancet Digit Health. 2023;5(6):e390-e4.

30. Dockendorf MF, Hansen BJ, Bateman KP, et al. Digitally Enabled, Patient-Centric Clinical Trials: Shifting the Drug Development Paradigm. Clin Transl Sci. 2021;14(2):445-59.

31. Mowlem FD, Tenaerts P, Gwaltney C, et al. Regulatory Acceptance of Patient-Reported Outcome (PRO) Data from Bring-Your-Own-Device (BYOD) Solutions to Support Medical Product Labeling Claims Let's Share the Success Stories to Move the Industry Forward. Therapeutic Innovation & Regulatory Science. 2022;56(4):531-5.

For peer review only

For peer review only - http://bmjopen.bmj.com/site/about/guidelines.xhtml

637 32. Di J, Demanuele C, Kettermann A, et al. Considerations to address missing data when deriving clinical trial endpoints from digital health technologies. Contemp Clin Trials. 2022;113:106661.

640 33. Curtis A, Qu Y. Impact of Using A Mixed Data Collection Modality on Statistical Inferences in Decentralized Clinical Trials. Ther Innov Regul Sci. 2022;56(5):744-52.

642 34. Idoko OT, Owolabi OA, Odutola AA, et al. Lessons in participant retention in the course of a randomized controlled clinical trial. BMC Res Notes. 2014;7:706.

644 35. Gunturu KS, Dizon DS, Johnson J, et al. Clinical trials in the era of digital engagement: a SWOG call to action. JCO Clin Cancer Inform. 2020;4:254-8.

646 36. Busisiwe N, Seeley J, Strode A, et al. Beyond translations, perspectives for researchers to consider to enhance comprehension during consent processes for health research in sub-saharan Africa: a scoping review. BMC Med Ethics. 2023;24(1):43.

649 37. Demasi M. FDA oversight of clinical trials is "grossly inadequate," say experts. BMJ (Clinical research ed). 2022;379:o2628.

651 38. FDA. Decentralized clinical trials for drugs, biological products, and devices - Guidance for industry, investigators, and other stakeholders: Silver Spring; 2020 [Available from: https://www.fda.gov/regulatory-information/search-fda-guidance-documents/decentralized-clinical-trials-drugs-biological-products-and-devices. Accessed 23.07.2023].

655 39. Deplanque D, Cviklinski S, Bardou M, et al. Health crisis: What opportunities for clinical drug research? Therapie. 2022;77(1):59-67.

657 40. Solarin O, Mohammed SI, Ndlovu N, et al. Partnerships and Collaborations: The Right Alliances for Clinical Trials in Africa. JCO Glob Oncol. 2020;6:954-8.

659 41. van Rijssel TI, de Jong AJ, Santa-Ana-Tellez Y, et al. Ethics review of decentralized clinical trials (DCTs): Results of a mock ethics review. Drug Discov Today. 2022;27(10):103326.

662 42. de Jong AJ, van Rijssel TI, Zuidgeest MGP, et al. Opportunities and Challenges for Decentralized Clinical Trials: European Regulators' Perspective. Clin Pharmacol Ther. 2022;112(2):344-52.

665

666 Figure Legend:

667 Figure 2 Challenges for decentralised clinical trials in sub Saharan Africa

For peer review only

For peer review only - http://bmjopen.bmj.com/site/about/guidelines.xhtml

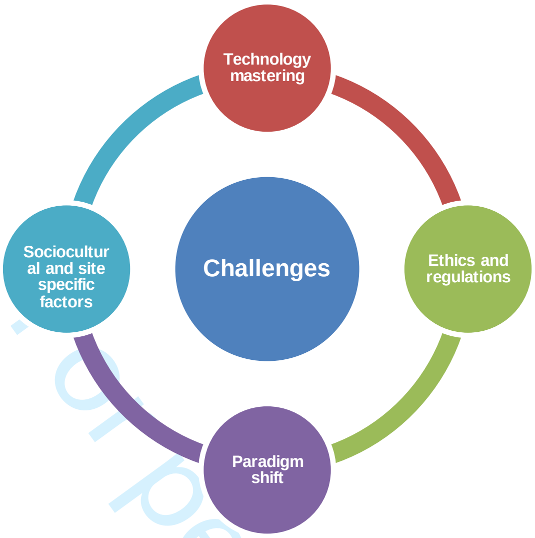

Figure 1: Challenges for decentralized clinical trials in sub-Saharan Africa

For peer review only

Standards for Reporting Qualitative Research (SRQR)*

http://www.equator-network.org/reporting-guidelines/srqr/

Page/line no(s).

Title and abstract

**Title** - Concise description of the nature and topic of the study Identifying the study as qualitative or indicating the approach (e.g., ethnography, grounded theory) or data collection methods (e.g., interview, focus group) is recommended	Page 1 Lines 1-2
**Abstract** - Summary of key elements of the study using the abstract format of the intended publication; typically includes background, purpose, methods, results, and conclusions	Page 2 Lines 16 - 41

Introduction

**Problem formulation** - Description and significance of the problem/phenomenon studied; review of relevant theory and empirical work; problem statement	Pages 4 - 5 Lines 57 – 96
**Purpose or research question** - Purpose of the study and specific objectives or questions	Page 5 Lines 94 - 96

Methods

**Qualitative approach and research paradigm** - Qualitative approach (e.g., ethnography, grounded theory, case study, phenomenology, narrative research) and guiding theory if appropriate; identifying the research paradigm (e.g., postpositivist, constructivist/ interpretivist) is also recommended; rationale**	Page 5- 6 Lines 114 - 139
**Researcher characteristics and reflexivity** - Researchers' characteristics that may influence the research, including personal attributes, qualifications/experience, relationship with participants, assumptions, and/or presuppositions; potential or actual interaction between researchers' characteristics and the research questions, approach, methods, results, and/or transferability	Page 6 - 7 Lines 155 - 174
**Context** - Setting/site and salient contextual factors; rationale**	Page 5 Lines 100 – 111
**Sampling strategy** - How and why research participants, documents, or events were selected; criteria for deciding when no further sampling was necessary (e.g., sampling saturation); rationale**	Page 5 Lines 114 - 139
**Ethical issues pertaining to human subjects** - Documentation of approval by an appropriate ethics review board and participant consent, or explanation for lack thereof; other confidentiality and data security issues	Page 17 Lines 538 - 545
**Data collection methods** - Types of data collected; details of data collection procedures including (as appropriate) start and stop dates of data collection and analysis, iterative process, triangulation of sources/methods, and modification of procedures in response to evolving study findings; rationale**	Pages 5 - 6 Lines 141 - 153

For peer review only - http://bmjopen.bmj.com/site/about/guidelines.xhtml

**Data collection instruments and technologies** - Description of instruments (e.g., interview guides, questionnaires) and devices (e.g., audio recorders) used for data collection; if/how the instrument(s) changed over the course of the study	Pages 5 - 6<br>Lines 122 - 153
**Units of study** - Number and relevant characteristics of participants, documents, or events included in the study; level of participation (could be reported in results)	Page 7<br>Lines 213 - 214
**Data processing** - Methods for processing data prior to and during analysis, including transcription, data entry, data management and security, verification of data integrity, data coding, and anonymization/de-identification of excerpts	Page 6<br>Lines 148 - 162
**Data analysis** - Process by which inferences, themes, etc., were identified and developed, including the researchers involved in data analysis; usually references a specific paradigm or approach; rationale**	Page 6<br>Lines 148 – 162
**Techniques to enhance trustworthiness** - Techniques to enhance trustworthiness and credibility of data analysis (e.g., member checking, audit trail, triangulation); rationale**	Page 6<br>Lines 155 – 174

Results/findings

**Synthesis and interpretation** - Main findings (e.g., interpretations, inferences, and themes); might include development of a theory or model, or integration with prior research or theory	Page 7 - 13<br>Lines 214 – 382
**Links to empirical data** - Evidence (e.g., quotes, field notes, text excerpts, photographs) to substantiate analytic findings	Page 8 – 13<br>Lines 222-223; 240-245; 252-254; 259-262; 267-270; 278-282; 287-291; 295-297; 311-313; 320-323; 328-329; 331-333; 337-340; 349-351; 358-363; 364-368; 372-373; 381-383

Discussion

**Integration with prior work, implications, transferability, and contribution(s) to the field -** Short summary of main findings; explanation of how findings and conclusions connect to, support, elaborate on, or challenge conclusions of earlier scholarship; discussion of scope of application/generalizability; identification of unique contribution(s) to scholarship in a discipline or field	Page 13 - 16<br>Lines 385 – 509
**Limitations** - Trustworthiness and limitations of findings	Page3; Page 16<br>45-56; 500 - 509

Other

For peer review only

For peer review only - http://bmjopen.bmj.com/site/about/guidelines.xhtml

**Conflicts of interest** - Potential sources of influence or perceived influence on study conduct and conclusions; how these were managed	Page 17 Lines 551 – 553
**Funding** - Sources of funding and other support; role of funders in data collection, interpretation, and reporting	Page 17 Lines 547 - 549

*The authors created the SRQR by searching the literature to identify guidelines, reporting standards, and critical appraisal criteria for qualitative research; reviewing the reference lists of retrieved sources; and contacting experts to gain feedback. The SRQR aims to improve the transparency of all aspects of qualitative research by providing clear standards for reporting qualitative research.

**The rationale should briefly discuss the justification for choosing that theory, approach, method, or technique rather than other options available, the assumptions and limitations implicit in those choices, and how those choices influence study conclusions and transferability. As appropriate, the rationale for several items might be discussed together.

Reference:
O'Brien BC, Harris IB, Beckman TJ, Reed DA, Cook DA. **Standards for reporting qualitative research: a synthesis of recommendations.** *Academic Medicine*, Vol. 89, No. 9 / Sept 2014 DOI: 10.1097/ACM.0000000000000388

For peer review only

For peer review only - http://bmjopen.bmj.com/site/about/guidelines.xhtml
